# Prediction of the Risk of Adverse Clinical Outcomes with Machine Learning Techniques in Patients with Noncommunicable Diseases

**DOI:** 10.1007/s10916-025-02140-z

**Published:** 2025-02-03

**Authors:** Alejandro Hernández-Arango, María Isabel Arias, Viviana Pérez, Luis Daniel Chavarría, Fabian Jaimes

**Affiliations:** 1 Department of Internal Medicine, University of Antioquia, Medellín, Colombia; 2 Hospital Alma Mater de Antioquia, University of Antioquia, Medellín, Colombia; 3 Health Information Systems Professional Living Lab. , Medellín, Colombia; 4 Data Scientist, National University, Medellín, Colombia; 5 Department of Internal Medicine, University of Antioquia, Medellín, Colombia; 6 Faculty of Medicine, Department of Internal Medicine, Hospital Alma Mater de Antioquia, University of Antioquia, University of Antioquia, Carrera 51 A # 62 – 42, Medellín, Colombia

**Keywords:** Clinical decision support system, Predictive models, Mortality, Emergency consultation, Hospitalization, Artificial intelligence

## Abstract

Decision-making in chronic diseases guided by clinical decision support systems that use models including multiple variables based on artificial intelligence requires scientific validation in different populations to optimize the use of limited human, financial, and clinical resources in healthcare systems worldwide. This cohort study evaluated three machine learning algorithms—XGBoost, Elastic Net logistic regression, and an Artificial Neural Network—to develop a prediction model for three outcomes: mortality, hospitalization, and emergency department visits. The objective was to build a clinical decision support system for patients with noncommunicable diseases treated at the Alma Mater Hospital complex in Medellín, Colombia. We collected 4845 electronic medical record entries from 5000 patients included in the study. The median age was 71.83 years, with 63.8% women and 29.7% receiving home care. The most prevalent medical conditions were diabetes (52.9%), hypertension (67.2%), dyslipidemia (57.3%), and COPD (19.4%). For mortality prediction, the Elastic Net logistic regression model achieved an AUCROC of 0.883 (95% CI: 0.848–0.917), the XGBoost model reached an AUCROC of 0.896 (95% CI: 0.865–0.927), and the Neural Network achieved 0.886 (95% CI: 0.853–0.916). For hospitalization, the Elastic Net model had an AUCROC of 0.952 (95% CI: 0.937–0.965), the XGBoost model achieved 0.963 (95% CI: 0.952–0.974), and the Neural Network scored 0.932 (95% CI: 0.915–0.948). For emergency department visits, the AUCROC values were 0.980 (95% CI: 0.971–0.987) for Elastic Net, 0.977 (95% CI: 0.967–0.986) for XGBoost, and 0.976 (95% CI: 0.968–0.982) for the neural network. A dashboard was developed to interact with an ensemble risk categorization segmenting patient risk in the cohort to aid in clinical decision-making. A clinical decision support system based on artificial intelligence using electronic medical records possibly can help segmenting the risk in populations with Noncommunicable Diseases for effective decision-making.

**Supplementary Information:**

The online version contains supplementary material available at 10.1007/s10916-025-02140-z.

## Introduction

 The increase in demand for health services from people with Noncommunicable Diseases represents a challenge for the health systems of many countries and ours is no exception. In Colombia, the high costs of chronic diseases are reflected in their diagnosis and treatment, which are characterized by being prolonged, complex and affecting the economically active population. Given that in many cases their diagnosis and intervention are late, in addition to the costs for the system, a burden is generated for the patient’s health and the stability of his family [1].

An appropriate strategy for this chronic disease problem may be to use a model of care based on risk stratification. Stratification is defined as “the identification and/ or grouping of patients according to risk or severity classification”, which serves to define in advance interventions that are tailored to their future health care needs (2). To carry out the stratification there are several systems of classification of patients, among which are: Adjusted Clinical *Groups (*ACG), a system that assigns each person in an exclusive category based on clinical criteria, with the aim of predicting health costs, pharmaceutical expenses and hospitalization risks [2]; *Diagnostic Cost* Groups (DCG), which use information from all diagnoses and prescriptions to form Clinical *Risk Groups* (CRG); which, together with demographic characteristics, manage to predict health costs in a year by classifying individuals according to the severity of their health status and according to their chronicity (4); and the Diagnosis-Related Groups (DRGs), which allow relating the different types of patients treated in a hospital, with the cost of their management. The use of DRGs is recommended by the World Health Organization (WHO) and several Latin American governments are exploring their implementation. However, this requires having information systems that have a high quality of the data, to guarantee the accuracy of the classifications and therefore the decisions that are made. In the case of Colombia, there has been evidence of low quality in the Individual Health Service Delivery Registries (RIPS) for the DRM system, as well as the absence of policies that promote and promote comparable health risk [3] (5).

The need to accurately direct the finite resources, both human and economic, of health systems towards a subpopulation at higher risk of adverse outcomes can be realized under the stratification of individual risk taken to population terms, which allows to reach a coordination of the level of care according to the presence of “clusters” or risk profiles in chronic diseases [4] Clinical decision support systems (CDS) can help clinicians make informed decisions if they are properly integrated into the treatment process, if they are easy to use and understand, and if they use standards that enable interoperability with other systems. If these CDS systems are designed and implemented with user needs in mind, they have the potential to improve medical decisions, streamline physicians’ work, and improve patient outcomes [5].

The hospital in which this research was carried out has developed a *“SerMás”* care model based on integrated and continuous care, promoting synergies in the health services network and co-management of health risk between the hospital and the insurer [6] Therefore, the aim of this study is to retrospectively derive a real-time risk prediction methodology for adverse clinical outcomes with machine learning techniques and *big data* analytics in patients with chronic Noncommunicable Diseases. The final goal is the creation of a prescriptive analysis dashboard as a clinical decision support system, which allows real-time interaction with predictions based on clinical and epidemiological characteristics of patients in the cohort.

## Methods

### Source of Data

A retrospective cohort study was conducted on electronic medical record records for the derivation of 2 prediction models of 3 outcomes: mortality, hospitalization and emergency room visit. Patient collection was conducted from April 1, 2017 to December 31, 2020 and outcome assessment from January 1 to December 31, 2020. This study was approved by the ethics committee of the Alma Mater Hospital in Antioquia (INS 2022-08). The data was always managed within the Hospital with security control and passwords in the work ecosystems to protect the identity of the patients. We followed the TRIPOD-AI consensus. [7]

### Participants

The study was carried out at a highly complex medical institution, Hospital Alma Mater de Antioquia, located in Medellín, Antioquia. This institution comprises an outpatient care facility, a home care division, and a hospital unit. Patients were eligible for inclusion if they were at least 18 years old and had at least one chronic disease as defined by the ICD-10 coding system [8] (Table 1 of supplementary appendix.). Patients were excluded if they lacked clinical data in their electronic medical records, often due to missed appointments or loss to follow-up.

**Table 1:** Clinical characteristics of patients included in the study cohort

Variable	Category	Data loss *n*(%)	Total *n* = 4845 (100%)
Age, Mean (SD)			71.8 (13.0)
Gender, n (%)	Female		3104 (64.1)
	Male		1741 (35.9)
Emergency room, n (%)			1029 (21.2)
Inpatients, n (%)			918 (18.9)
Surgery, n (%)			370 (7.6)
General practitioner, n (%)			2832 (58.5)
Specialized medicine, n (%)			3464 (71.5)
General wards LoS Days, Mean (SD)			7.8 (9.7)
Special Care LoS Days, Mean (SD)			4.5 (3.5)
Intensive Care ICU LoS Days, Mean (SD)			7.2 (7.7)
Depression and Mood Disturbances, n (%)			990 (20.4)
COPD, n (%)			940 (19.4)
Thyroid diseases, n (%)			808 (16.7)
Somatoform, n (%)			808 (16.7)
Osteoarthritis, n (%)			798 (16.5)
Ischemic heart disease, n (%)			789 (16.3)
Chronic Kidney Disease, n (%)			660 (13.6)
Obesity, n (%)			566 (11.7)
Heart Failure, n (%)			545 (11.2)
Cerebrovascular, n (%)			472 (9.7)
Dementia, n (%)			443 (9.1)
Osteoporosis, n (%)			424 (8.8)
Atrial Fibrillation, n (%)			350 (7.2)
Sleep Disorders, n (%)			345 (7.1)
Hypertension, n (%)			3267 (67.4)
Vertigo and Hearing Impairment, n (%)			286 (5.9)
Other Genitourinary, n (%)			282 (5.8)
Venous and Lymphatic Diseases, n (%)			282 (5.8)
Peripheral Neuropathies, n (%)			271 (5.6)
Upper Gastrointestinal Diseases, n (%)			268 (5.5)
Migraine and Painful Facial Syndromes, n (%)			251 (5.2)
Colitis and Lower Gastrointestinal, n (%)			248 (5.1)
Prostate Diseases, n (%)			222 (4.6)
Epilepsy, n (%)			213 (4.4)
Diabetes, n (%)			2123 (43.8)
Dyslipidemia, n (%)			2057 (42.5)
Anemia, n (%)			136 (2.8)
Weight, Mean (SD)		617(12.71%)	68.1 (15.0)
Height, Mean (SD)		617(12.71%)	156.2 (9.3)
Thigh Circumference, Mean (SD)		617(12.71%)	48.3 (94.2)
Waist Circumference, Mean (SD)		617(12.71%)	95.6 (17.5)
triceps fold measurement, Mean (SD)		617(12.71%)	17.7 (15.4)
Abdomen Fold measure, Mean (SD)		617(12.71%)	26.5 (86.6)
Thigh Fold, Mean (SD)		617(12.71%)	21.7 (15.2)
Systolic Blood Pressure, Mean (SD)		617(12.71%)	129.8 (20.4)
Diastolic Blood Pressure, Mean (SD)		617(12.71%)	73.4 (11.2)
Resting Heart Rate, Mean (SD)		617(12.71%)	76.0 (11.9)
Self-rated Exercise level, n (%)	1.0	617(12.71%)	4144 (98.0)
	2.0		24 (0.6)
	3.0		16 (0.4)
	4.0		15 (0.4)
	5.0		29 (0.7)
METS metabolic rate, Mean (SD)		617(12.71%)	4.8 (2.5)
VO2 at maximum oxygen, Mean (SD)		617(12.71%)	17.0 (8.6)
Gröningen Fragility Index, n (%)	Fragile		1968 (40.6)
	Data Not Available	617 (12.7)	
	Normal		2260 (46.6)
Monopodial time, Mean (SD)		617(12.71%)	7.8 (10.3)
Ankle-Brachial Mndex, n (%)	0.41 to 0.90		70 (1.4)
	0.91 to 1.30		1574 (32.5)
	< 0.4		33 (0.7)
	Not qualified		3168 (65.4)
Blood Glucose, Mean (SD)		944(19,45%)	102.3 (118.6)
Glycated Haemoglobin HbA1c, Mean (SD)		944(19,45%)	5.0 (3.3)
LDL, Mean (SD)		944(19,45%)	47.2 (48.0)
HDL, Mean (SD)		944(19,45%)	41.6 (159.8)
Total Cholesterol, Mean (SD)		944(19,45%)	134.0 (68.3)
Triglycerides, Mean (SD)		944(19,45%)	125.3 (92.2)
Framingham Cardiovascular Risk adjusted to Colombia, n (%)	High risk		1710 (35.3)
	Low risk		2177 (44.9)
	Not rated		958 (19.8)
Glomerular Filtration Rate (GFR), Mean (SD)		944(19,45%)	59.9 (39.7)
Stage of Chronic Kidney Disease (CKD), n (%)	Stage 0		1511 (31.2)
	Stage 1		466 (9.6)
	Stage 2		1029 (21.2)
	Stage 3a		796 (16.4)
	Stage 3b		720 (14.9)
	Stage 4		253 (5.2)
	Stage 5		70 (1.4)
Urinary Albumin to Creatinine Ratio, Mean (SD)		944(19,45%)	44.3 (271.4)
TSH, Mean (S)		944(19,45%)	2.6 (6.6)
*Functional Classification by “SerMás”*, n (%)	Functional class 1		39 (0.8)
	Functional class 2A		1482 (30.6)
	Functional class 2B		814 (16.8)
	Functional class 3		145 (3.0)
	Functional class 4		1421 (29.3)
	Not rated		944 (19.5)

*n (%)*: Number and percentage of participants, SD: Standard Deviation, *LoS*: Length of Stay,* ICU*: Intensive Care Unit, *METS*: metabolic equivalents, *V̇O*: Maximal Oxygen Consumption, *HbA1c*: Glycated Hemoglobin, *COPD*: Chronic Obstructive Pulmonary Disease, *TSH*: Thyroid-Stimulating Hormone, *CKD*: Chronic Kidney Disease, *GFR*: Glomerular Filtration Rate, *HDL*: High-Density Lipoprotein, *LDL*: Low-Density Lipoprotein *Additional results on Hartigan immersion test, normality test and Tukey’s test are described in Supplementary appendix

Patient care followed the protocol of the *“SerMás”* care model, a comprehensive health management approach coordinating efforts between different health services. Importantly, the study utilized a convenience sampling method based on a contract with the healthcare payer. The cohort consisted of 5,000 patients selected in advance by the insurer according to the inclusion criteria.

### Outcomes

1. Hospital and out-of-hospital mortality: data were obtained from the GHIPS system (2024 “ALMA MATER HOSPITAL” Version: 31.2.20221216 to 37) and out-of-hospital mortality was confirmed by the health insurer and the RUAF (©information system that consolidates the affiliations reported by the entities and administrators of the Social Protection System in Colombia).
2. Hospitalization: data were obtained on the number of times the patient consulted the assigned referral hospital or other hospitals the data was reported to the hospital when the patient was hospitalized elsewhere.
3. Use of emergency only in the reference hospital.

### Predictor Variables

We included 164 variables which were obtained from the GHIPS system, a web application that works as an electronic medical record system of the hospital in the outpatient and hospital settings. Clinical, laboratory and billing variables were extracted (Table 2 of supplementary appendix). In addition, the outcomes of mortality, hospitalization and emergency use during the evaluation year were obtained.

**Table 2:** Statistical analysis of Artificial neural network, XGBoost and Elastic net logistic regression models for mortality, hospitalization, and emergency room consultation

Outcome	Model name	Sensitivity (Recall)	Specificity (Relectivity)	Positive Predictive Value (Precision)	Negative Predictive Value	AUCROC	Intercept	Slope
Hospitalization	*Elastic Net*	0.683 (0.630–0.733)	0.974 (0.965–0.983)	0.881 (0.839–0.922)	0.916 (0.900–0.932)	0.952 (0.937–0.965)	−3.110 (−3.343–2.914)	6.406 (5.875–7.030)
	*XGBoost*	0.792 (0.746–0.838)	0.955 (0.943–0.968)	0.833 (0.789–0.878)	0.942 (0.928–0.956)	0.963 (0.952–0.974)	−3.526 (−3.799–3.308)	6.769 (6.299–7.332)
	*Neural Network*	0.581 (0.526–0.635)	0.980 (0.972–0.988)	0.893 (0.850–0.933)	0.892 (0.874–0.909)	0.932 (0.915–0.948)	−2.679 (−2.867–2.507)	6.048 (5.484–6.747)
Emergency Room Consultation	*Elastic Net*	0.927 (0.897–0.952)	0.964 (0.951–0.974)	0.889 (0.852–0.919)	0.977 (0.967–0.985)	0.980 (0.971–0.987)	−3.778 (−4.074–3.516)	6.575 (6.125–7.068)
	*XGBoost*	0.905 (0.874–0.936)	0.961 (0.950–0.972)	0.878 (0.845–0.913)	0.970 (0.960–0.980)	0.977 (0.967–0.986)	−3.792 (−4.046–3.564)	7.212 (6.765–7.744)
	*Neural Network*	0.810 (0.762–0.850)	0.963 (0.952–0.974)	0.872 (0.834–0.909)	0.942 (0.926–0.956)	0.976 (0.968–0.982)	−3.019 (−3.265–2.794)	5.476 (5.070–5.939)
Mortality	*Elastic Net*	0.336 (0.259–0.414)	0.991 (0.985–0.996)	0.807 (0.702–0.906)	0.931 (0.918–0.943)	0.883 (0.848–0.917)	−3.275 (−3.489–3.079)	6.523 (5.778–7.240)
	*XGBoost*	0.453 (0.364–0.531)	0.986 (0.980–0.992)	0.785 (0.690–0.864)	0.942 (0.929–0.954)	0.896 (0.865–0.927)	−3.353 (−3.593–3.146)	6.311 (5.692–6.952)
	*Neural Network*	0.358 (0.275–0.437)	0.985 (0.979–0.992)	0.731 (0.627–0.839)	0.933 (0.919–0.945)	0.886 (0.853–0.916)	−3.023 (−3.235–2.820)	5.601 (4.894–6.414)

*Sensitivity (Sens) is the ability of the model to correctly rule out patients with the outcome of interest. ^Specificity (Spec) is the ability of the model to correctly detect patients without the outcome of interest. ¨Negative predictive value (NPV) is the probability that a patient will not have the outcome of interest if the model classifies it as such. +Positive predictive value (PPV) is the probability that a patient will have the outcome of interest if the model classifies it as such AUCROC (Area under the ROC curve) is a numerical measure that evaluates the model’s ability to differentiate between patients with and without the outcome of interest. 95% CI was calculated

### Sample Size Calculation

A formal sample size calculation was not made because there was a fixed cohort.

### Imputation of Missing Data

A data imputation process was performed to variables with less than 20% data losses using the K Nearby Neighbors (KNN) algorithm, an imputation technique that uses information from existing data to estimate missing values. The algorithm selects a number k of observations closest to the observation with the missing value (neighbors) in the complete dataset. Then use the mean of those k neighbors to estimate the missing value. [9]

### Statistical Analysis

For normally distributed variables, the mean and standard deviation are usually shown, while variables that are not normally distributed are reported with the median and interquartile range. The Hartigan immersion test was applied to describe possible multimodal distributions and the Tukey test to describe variables with distant outliers [10] DeLong’s Test, was used to define differences between model’s areas under the curve. All statistical measures were calculated with accompanying 95% confidence intervals (CIs) and w used a p-value threshold of 0.05.

### Models

Three supervised learning models were used: Elastic Net logistic regression model, an Artificial Neural Network and the XGBoost algorithm. For the 3 outcomes, the database was divided into 2 parts: 85% for model training and 15% for a test dataset (Internal Validation). Before entering the data into the machine learning models, the numerical variables were centered at a mean of zero and then scaled to ensure that they all have a variance of 1. For the nominal variables, *Dummies* (indicator) variables (12) [11] with the ICD reference category 10 described in Table 1 of the supplementary appendix.

The Elastic Net logistic regression model is an extension of the traditional logistic regression model that uses regularization techniques to reduce the risk of overfitting, and uses a combination of the vector norm L1 (the sum of the absolute value of the elements of the vector) and L2 (Euclidean norm is the square root of the sum of the squares of the elements of the vector) for regularization, what is known as Lasso (L1), Ridge (L2) and Elastic Net (L1 and L2) regularization respectively, to automatically select the most important characteristics in the data and avoid overfitting [12].

The XGBoost algorithm is an implementation of *gradient boosting* with decision trees, Gradient *boosting* is a machine learning technique that consists of training a set of decision trees sequentially, where each tree is trained to correct the errors of the previous tree. XGBoost uses a gradient optimization technique called “stochastic gradient regularization” to adjust the parameters of individual decision trees [13].

In the neural network architecture, a feedforward model was implemented using the Keras framework. The network consisted of an input layer corresponding to the number of features in the dataset, followed by three hidden layers with 128, 64, and 32 neurons, respectively, each using the ReLU activation function. Batch normalization was applied after each hidden layer to standardize inputs and improve training stability, while a dropout rate of 0.5 was used for regularization to prevent overfitting. The output layer included a single neuron with a sigmoid activation function for binary classification. The model was optimized using the Adam optimizer with a learning rate of 0.001 and the binary cross-entropy loss function, while accuracy was tracked as a performance metric. Training was performed over 100 epochs with a batch size of 32, and early stopping was employed to terminate training when validation performance plateaued. Additional callbacks, including model checkpointing, TensorBoard logging, and a custom callback to monitor epoch-wise training times, were utilized to enhance training efficiency and transparency. Model architecture shown in suplementary sFigure 6.

For the three models all the hyperparameters were initialized randomly, a set of fitting data of these “hyperparameters” was not used and instead the 10-fold cross-validation technique was performed for each of the three models with the three outcomes, to find the best parameters between the training dataset and the validation dataset. [14] The metrics area under receiver operating characteristic curve (AUCROC), sensitivity, specificity, negative predictive value, positive predictive value and calibration curves were determined with the calculation of the slope and intercept for each outcome. For each metric, the 95% confidence interval was calculated and a maximum alpha error of 0.05 was accepted.

Models were selected for each outcome with better discrimination in AUCROC and no statistically significant differences in slope and intercept in calibration curve. The results were compared using the DeLong test for differences in the AUCROC of each of the outcomes [15].

The R programming language (version 4.2.2 Copyright (C) 2022 The R Foundation for Statistical Computing) and Python (Python Software Foundation (2021) were used. Python Language Reference, version 3.10.) to process the data and derive the model.

### Risk Groups

**Fig. 1:**
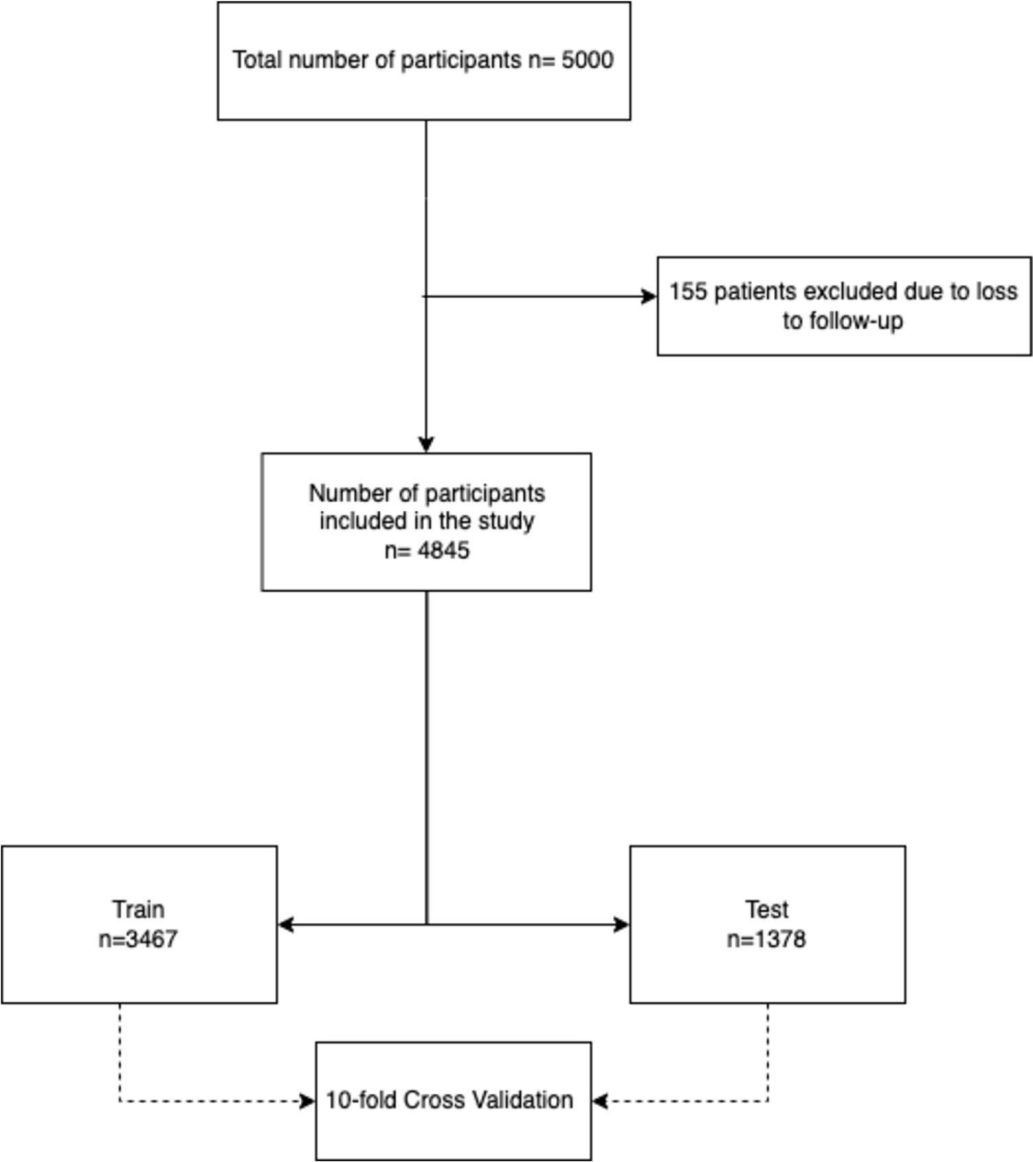
Patient flowchart in the study

Risk groups were created through a demo dashboard in Tableau software (Tableau. (2021). Tableau 2021.2 [Software]), in which the AI model was connected to make predictions and visualize the results in histogram form in the cohort. This allows patients to be displayed and filtered in the context of their prediction, to support decisions about the use of clinical resources and prioritization according to their risk. The dashboard creates histograms to predict the 3 outcomes with the probability extracted from the model on the X-axis and the number of patients on the Y-axis. This dashboard is the input for the end user to interact with the predictions (Fig. 1).

## Results

**Fig. 2:**
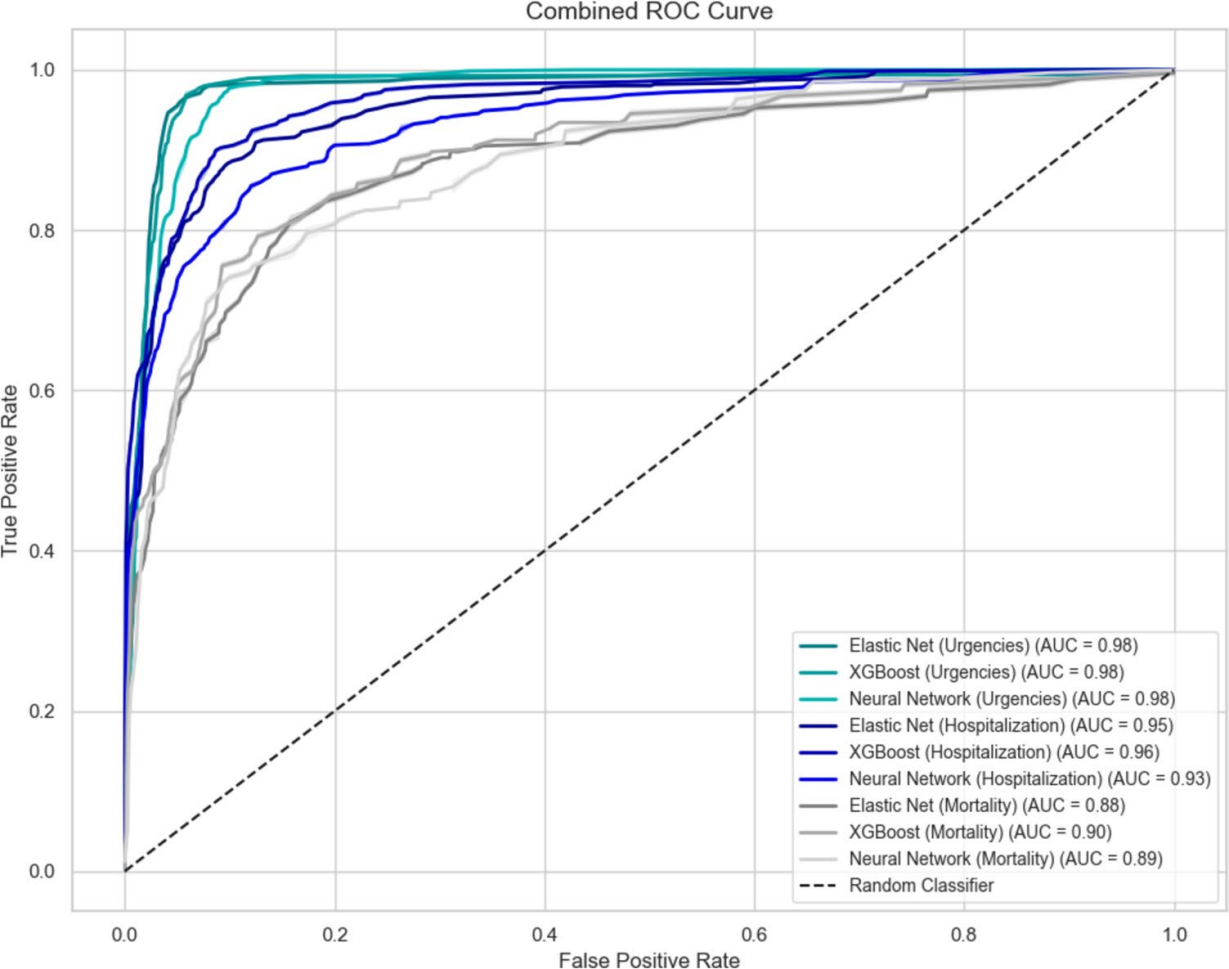
Area under receiver operating characteristic curve (AUCROC) of Artificial Neural Network, Elastic Net and XGBoost models for Hospitalization, Mortality and Emergency Room Consultation outcomes

Data were collected from January 2020 to December 2020 for a total of 5000 eligible patients and 4845 finally analyzed (Fig. 2). The cohort had a mean age of 71.8 years (standard deviation of 13.0) with 64.1% (*n* = 3104) women. 21.2% (*n* = 1029) of patients presented to the emergency department and 18.9% (*n* = 918) were hospitalized. 58.5% (*n* = 2832) consulted a general practitioner and 71.5% (*n* = 3464) consulted a specialist physician. The most common comorbidities were hypertension (67.4%), diabetes (43.8%) and dyslipidemia (42.5%). 19.4% of patients had chronic obstructive pulmonary disease (COPD), 16.7% had thyroid disease and 11.2% heart failure. The total mean value of billing in Colombian pesos was COP 5,468,904 per patient in the year (standard deviation of 7,376,458) (Table 1.) The distribution of chronic disease categories is presented in Table 3 of the Supplementary Appendix.

## Models

**Fig. 3:**
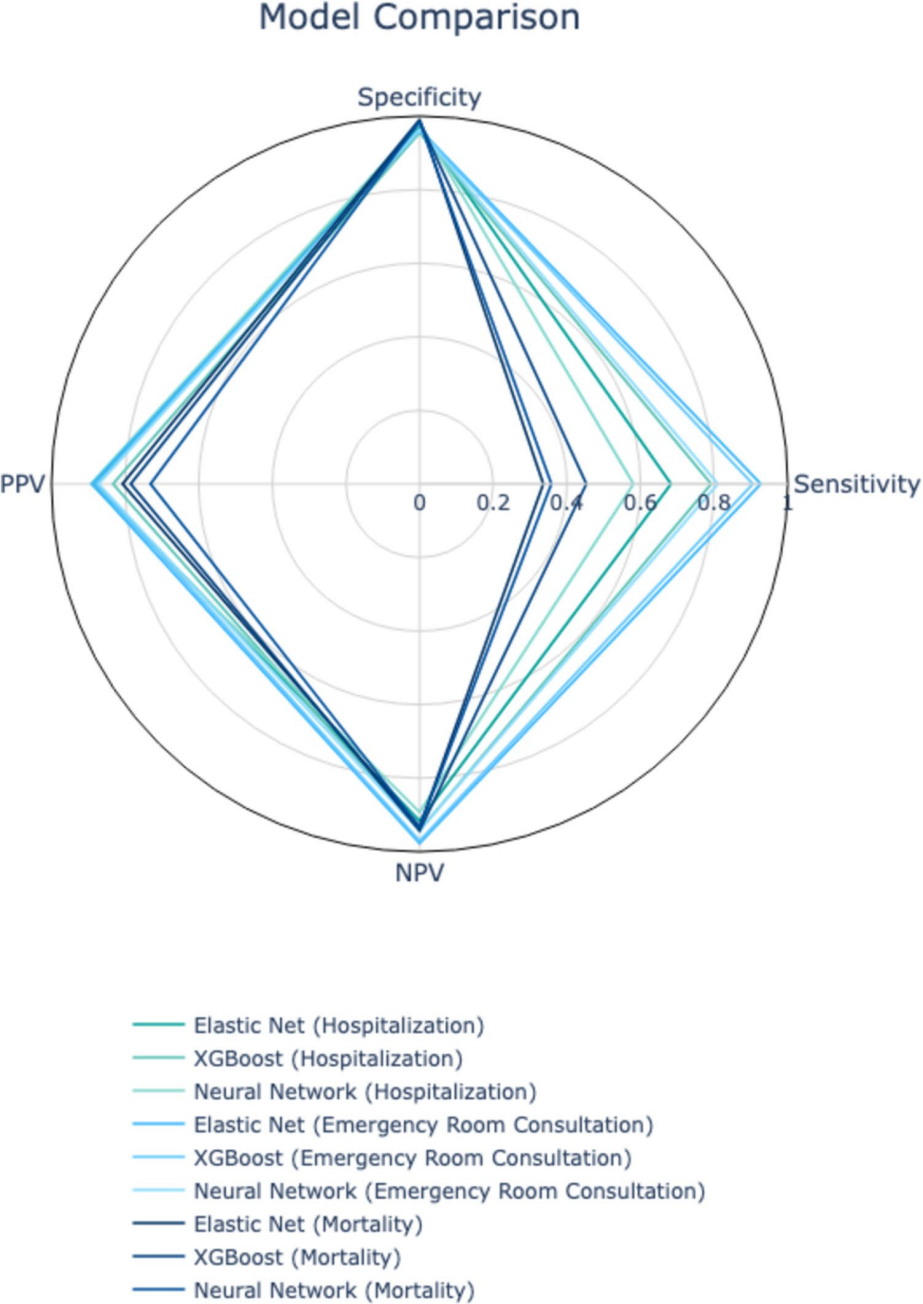
Spider plot of Artificial Neural Network, Elastic Net and XGBoost models for Hospitalization, Mortality and Emergency Room Consultation metrics

For the mortality outcome, the Elastic Net logistic regression model achieved an AUCROC of 0.883 (95% CI: 0.848–0.917), while the XGBoost model had an AUCROC of 0.896 (95% CI: 0.865–0.927). The neural network model performed similarly with an AUCROC of 0.886 (95% CI: 0.853–0.916). For the hospitalization outcome, Elastic Net showed an AUCROC of 0.952 (95% CI: 0.937–0.965), XGBoost reached 0.963 (95% CI: 0.952–0.974), and the neural network model achieved 0.932 (95% CI: 0.915–0.948). Regarding emergency room consultations, the AUCROC values were 0.980 (95% CI: 0.971–0.987) for Elastic Net, 0.977 (95% CI: 0.967–0.986) for XGBoost, and 0.976 (95% CI: 0.968–0.982) for the neural network model. (Fig. 3).

**Fig. 4:**
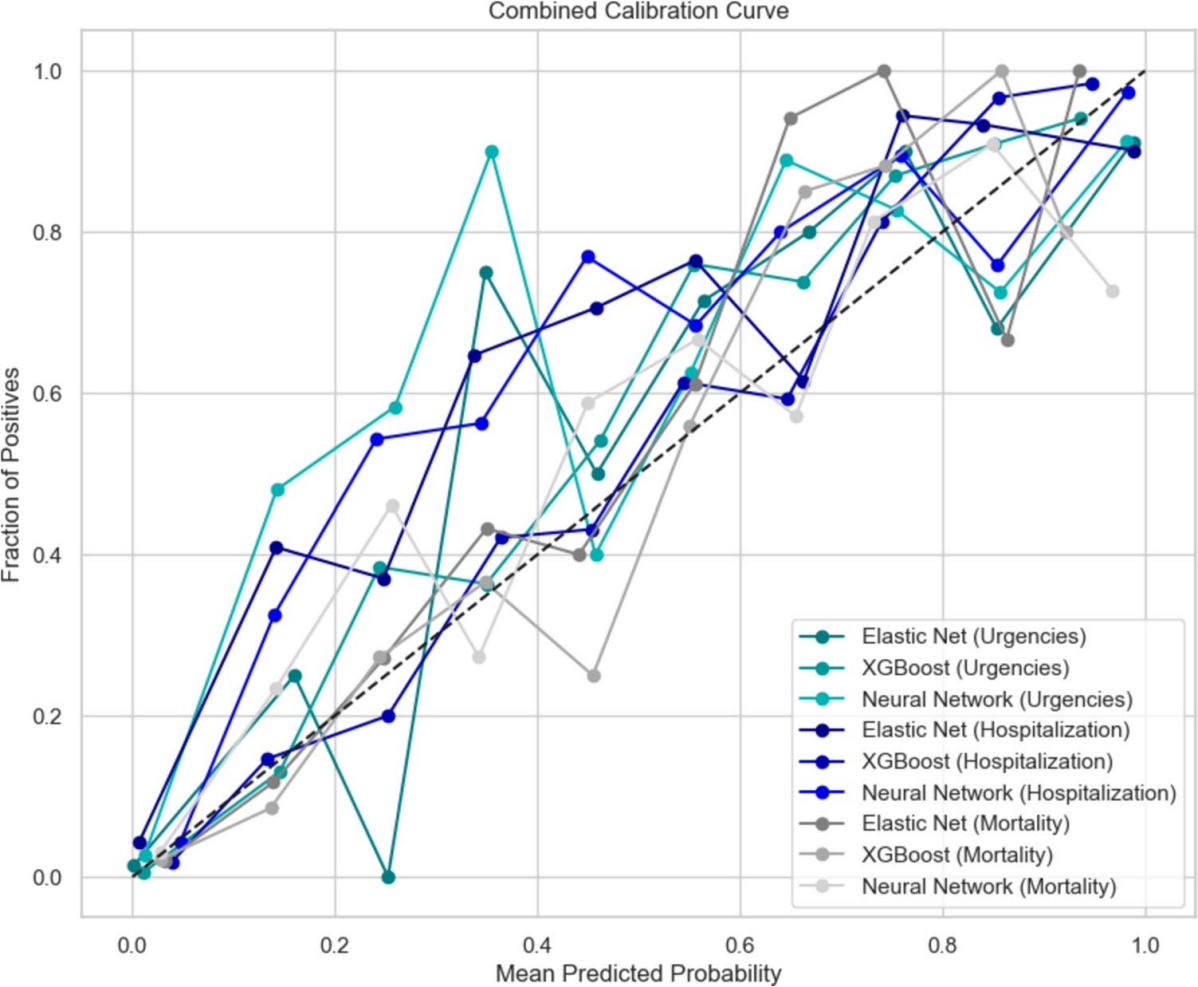
Calibration plots of Artificial Neural Network, Elastic net Logistic Regression and XGBoost models for Hospitalization, Mortality and Emergency Room Consultation outcomes

**Fig. 5:**
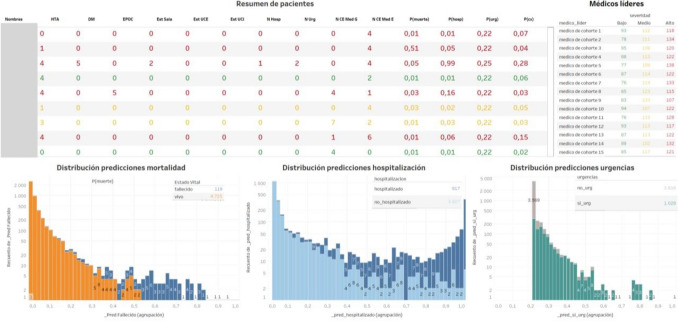
User friendly dashboard to interact with the prediction of models for Hospitalization, Mortality and Emergency Room Consultation for each patient metrics

In Table 2 we show the summary of all metrics performance of the three prediction models for the three selected outcomes. For mortality prediction, Elastic Net logistic regression achieved a sensitivity of 33.6% and a specificity of 99.1%. XGBoost outperformed Elastic Net with a sensitivity of 45.3% and specificity of 98.6%, while the neural network exhibited similar performance with a sensitivity of 35.8% and specificity of 98.5%. For hospitalization prediction, Elastic Net achieved a sensitivity of 68.3% and specificity of 97.4%, XGBoost reached a sensitivity of 79.2% and specificity of 95.5%, and the neural network demonstrated a sensitivity of 58.1% and specificity of 98.0%. Lastly, for emergency room consultations, Elastic Net exhibited high specificity (96.4%) but a lower sensitivity of 92.7%. XGBoost showed a balance of sensitivity (90.5%) and specificity (96.1%), and the neural network model achieved sensitivity and specificity of 81.0% and 96.3%, respectively. Figure 4 presents a spider plot comparing the performance of the nine models across multiple metrics. The calibration of these models against the outcomes is illustrated in Fig. 5, The weighting of the primary variables for each model and their contributions to the three outcomes are detailed in the Supplementary Appendix.

## Discussion

There are several limitations in the study that must be considered. First, the retrospective observational design, since the study represents the first step in the derivation of a risk model for the creation of a clinical decision support system within the framework of the DECIDE AI consensus [16] Therefore, more prospective research with intervention studies is needed to validate the model in different populations and healthcare settings before it can be used in clinical practice. Second, the study was conducted in a single highly complex reference hospital with an elderly population with multiple chronic diseases, without adequate representation of young patients. Although the hospital had different settings of home, outpatient and hospital care, there were differences in the balance of demographic characteristics such as the marked difference between women and men. No race information was collected, which could limit generalizability of results to other hospitals or healthcare settings. Third, the sample was very small relative to the amount recommended in studies employing machine learning approaches [17] and included a large number of predictive variables, making the assembly process with other types of medical history software technically difficult. Fourth, the sample size was predetermined by the insurer based on contractual convenience rather than a formal calculation of statistical power. While this cohort provided a substantial data for initial modeling, the lack of randomization or deliberate design in the sample selection could introduce biases and limit the ability to generalize findings. Future studies should aim to evaluate the model on larger and more diverse populations with sample sizes informed by power analyses.

In this study, prediction models for mortality, hospitalization, and emergency room visits were developed using Elastic Net Logistic Regression, XGBoost, and an Artificial Neural Network (ANN). The DeLong test revealed statistically significant differences in AUCROC favoring XGBoost over Elastic Net for hospitalization (*p* < 0.001), while Elastic Net outperformed the ANN (*p* = 0.008). For mortality, no significant differences were observed between Elastic Net, XGBoost, and the ANN (*p* > 0.05). Similarly, for emergency room consultations, no significant differences were found across all models (*p* = 1). These results emphasize the comparative performance advantages of XGBoost for certain outcomes while highlighting similar performance across models in others (Supplementary Table 4). For the calibration of the emergency room consultation outcome, both models showed overestimation; however, the Elastic Net regression model exhibited a significantly higher slope (12.23; 95% CI: 10.64–13.83) compared to XGBoost (1.2; 95% CI: 1.07–1.34). For hospitalization, Elastic Net regression showed no significant underestimation of risk. Across the remaining calibration comparisons, no significant differences were observed. Previously, in the same cohort, a functional scale for predicting mortality (C-statistical of 0.721 95% CI: 0.660–0.780), emergency room (C-statistical of 0.570 95% CI: 0.500–0.640) and hospitalization (C-statistical of 0.609 CI95%: 0.570–0.650) was developed and validated, so this study presents a predictive approach of greater discrimination of adverse outcomes of the *“SerMás”* cohort (8). [16]

While the models performed well overall in our study, XGBoost performed better. This same finding has been observed in another research. Forrest et al. derived and validated a model of random decision trees to predict coronary heart disease with an AUROC of 0.95 (95% CI 0.94 to 0.95), a sensitivity of 0.94 (95% CI 0.94 to 0.95) and a specificity of 0.82 (95% CI 0.81 to 0.83) (19). Li et al. evaluated the ability of XGBoost and logistic regression and other algorithms to predict mortality in heart failure patients admitted to the ICU. The results showed that XGBoost and logistic regression lasso L1 with AUCROC of 0.8416 (95% CI 0.7864 to 0.8967) had a superior performance compared to the risk score model “The *American Heart Association Get With The Guidelines a Heart Failure GWTG - HF”*, which exhibited an AUCROC of 0.7856 (95% CI 0.7183 to 0.8470). However, the XGBoost showed a wide net profit threshold range (> 0.1) above the other two models [18] Another study to predict ICU admission from the emergency room found that XGBoost performed well compared to deep neural networks (DNNs). The XGBoost model obtained an AUCROC of 0.861 (95% CI 0.848 to 0.874) with a higher discriminative yield than the DNN model with an AUCROC of 0.833 (95% CI 0.819 to 0.848) [19] Khera et al., compared the performance of some artificial intelligence algorithms, including XGBoost against logistic regression, in predicting mortality in patients with acute myocardial infarction. It found that the XGBoost model reclassified 32,393 of 121,839 patients (27%) at moderate to high risk of death, considered to be low risk in the logistic regression model [20].

This study highlights the predictive power of billing administrative variables for identifying clinical outcomes, such as mortality, emergency visits, and hospitalizations. These outcomes serve as proxies for underlying patient risk categories, enabling clinicians to stratify risk and allocate resources more effectively. For example, MacKay et al. developed a machine learning model combining administrative and clinical data to predict 30-day mortality with an AUROC of 0.88 using XGBoost, compared to 0.84 for logistic regression. Their model provided an interactive interface for clinicians to manage risk [21], like the approach implemented in this study (Fig. 1).

**Fig. 6:**
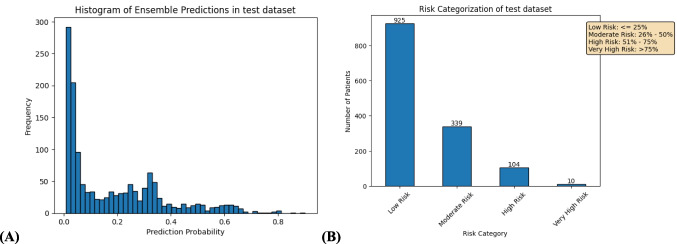
Ensemble Model-Based Risk Stratification (XGBoost): (**A**) Distribution of Prediction Probabilities and (**B**) Patient Risk Categorization of adverse clinical outcome

In our analysis, XGBoost demonstrated superior sensitivity, meaning it is better at identifying patients truly at risk for adverse outcomes. This makes its negative predictions more reliable, crucial for minimizing missed risks. Additionally, XGBoost’s sensitivity and calibration position it as a strong candidate for ensemble learning. Figure 6 illustrates risk stratification based on ensemble predictions, where patients are categorized into actionable risk levels.

To translate this predictive model into clinical impact, future work will focus on conducting a randomized controlled trials to evaluate interventions driven by this risk stratification approach.

## Conclusions

In conclusion, the XGBoost model presented a better performance than artificial neural networks, logistic regression and Elastic Net. Overall, the results indicate that the XGBoost model has the potential to be a tool for building clinical decision support systems that function as useful prognostic models for decision-making in patients with Noncommunicable Diseases. These types of tools should be evaluated and validated in future experimental studies for safe implementation in clinical flowcharts.

## Supplementary Information

Below is the link to the electronic supplementary material.

ESM1 (DOCX 1.90 MB)

## Data Availability

The data that support the findings of this study are available from the authors, but restrictions apply to the availability of these data, which were used under ethical approval from Hospital Alma Máter de Antioquia for the current study, and so are not publicly available. Data are, however, available from the authors upon reasonable request and with permission from the Hospital Alma Máter de Antioquia.

## Declarations

The study was approved by the Ethics Committee on Human Research at the University Research Unit (CBE-SIU), Universidad de Antioquia, under number 20-114-922. The Committee deemed the retrospective observational study ethically valid and posing no risk to participants. The Committee had ongoing access to study data while maintaining confidentiality and approved the measures for participant protection and the informed consent process. The study was conducted in accordance with Resolution 008430 of October 4, 1993, of the Colombian Ministry of Health, which established the scientific, technical, and administrative standards for health research; the principles of the World Medical Assembly as stated in the Declaration of Helsinki of 1964, last updated in 2013; the Code of Federal Regulations, Title 45, Part 46, for the protection of human subjects by the U.S. Department of Health and Human Services and the National Institutes of Health (June 18, 1991); and Resolution 2378 of 2008 of the Ministry of Social Protection of Colombia, which adopts Good Clinical Practices for institutions conducting research with medications in humans.
