# Supplementary material for: Prediction of the Risk of Adverse Clinical Outcomes with Machine Learning Techniques in Patients with Noncommunicable Diseases
Source: J Med Syst. 2025 Feb 3;49(1):19. doi: 10.1007/s10916-025-02140-z (PMC11790785; doi:10.1007/s10916-025-02140-z)

**Supplementary Appendix: Prediction of the risk of adverse clinical outcomes with machine learning techniques in patients with chronic no communicable diseases**

Alejandro Hernández-Arango MD. MS. ^1,2^ María Isabel Arias ^3,4^ ,Viviana Pérez ^2^, Luis Daniel Chavarría ^3^ , Fabian Jaimes phD.^4.^

^1^ Professor Department of Internal Medicine, University of Antioquia, School of Medicine Medellin, Colombia

^2^ Hospital Alma Máter de Antioquia, University of Antioquia. Medellin, Colombia.

^3^ Data Scientist, National University. Medellin, Colombia.

^4^ Full Professor, Department of Internal Medicine, University of Antioquia, Medellín, Colombia

*Other results of Statistical tests*

The Hartigan immersion test reports possible multimodal distributions for: age, room stay, ICU stay, total value, weight, height, oxygen saturation, thigh circumference, waist circumference, triceps fold, abdomen fold, thigh fold, systolic blood pressure, diastolic blood pressure, resting heart rate, monopodal seconds time, time to diagnosis, glycemia, glycated hemoglobin, how many cigarettes per day, lipoprotein, HDL, total cholesterol, Triglycerides, creatinine 1 consultation, glomerular filtration rate GFR, months difference between GFR, microalbuminuria, TSH.

The normality test reports non-normal distributions for: age, ward stay, ICU stay, ICU stay, total value, weight, height, oxygen saturation, thigh circumference, waist circumference, triceps fold, abdomen fold, thigh fold, systolic blood pressure, diastolic blood pressure, resting heart rate, mets -metabolic index, VO2 - maximum amount of oxygen, monopodal seconds time, time to diagnosis, glycemia, glycated hemoglobin, how many cigarettes per day, lipoprotein, HDL, total cholesterol, triglycerides, creatinine 1 consultation, glomerular filtration rate GFR, months difference between GFR, microalbuminuria, TSH.

Tukey's test indicates distant outliers in: ward stay, ICU stay, total value, weight, height, oxygen saturation, thigh circumference, waist circumference, triceps fold, abdomen fold, systolic blood pressure, diastolic blood pressure, resting heart rate, METS -metabolic index, VO2 - maximum amount of oxygen, glycemia, glycated hemoglobin, how many cigarettes per day, HDL, total cholesterol, triglycerides, creatinine 1 consultation, glomerular filtration rate GFR, months difference between GFR, microalbuminuria, TSH.

**Supplementary Table s1. Descriptors of ICD-10 codes included and excluded in each chronic disease category.**

| **ALLERGY** | |
| --- | --- |
| **Included ICD-10 codes and labels** | |
| J301 | Allergic rhinitis due to pollen |
| J302 | Other seasonal allergic rhinitis |
| J303 | Other allergic rhinitis |
| J304 | Allergic rhinitis, unspecified |
| J450 | Predominantly allergic asthma |
| K522 | Allergic and dietetic gastroenteritis and colitis |
| L20 | Atopic dermatitis |
| L23 | Allergic contact dermatitis |
| L500 | Allergic urticaria |
| Z516 | Desensitization to allergens |
| **ANEMIA** | |
| **Included ICD-10 codes and labels** | |
| D50 | Iron deficiency anaemia |
| D51 | Vitamin B12 deficiency anaemia |
| D52 | Folate deficiency anaemia |
| D53 | Other nutritional anaemias |
| D55 | Anaemia due to enzyme disorders |
| D56 | Thalassaemia |
| D57 | Sickle-cell disorders |
| D58 | Other hereditary haemolytic anaemias |
| D59 | Acquired haemolytic anaemia |
| D60 | Acquired pure red cell aplasia [erythroblastopenia] |
| D61 | Other aplastic anaemias |
| D63 | Anaemia in chronic diseases classified elsewhere |
| D64 | Other anaemias |
| **Excluded ICD-10 codes and labels** | |
| D563 | Thalassemia screen |
| D590 | Drug-induced autoimmune haemolytic anaemia |
| D592 | Drug-induced nonautoimmune haemolytic anaemia |
| D593 | Haemolytic-uraemic syndrome |
| D596 | Haemoglobinuria due to haemolysis from other external causes |
| D601 | Transient acquired pure red cell aplasia |
| D611 | Drug-induced aplastic anaemia |
| D612 | Aplastic anaemia due to other external agents |
| D642 | Secondary sideroblastic anaemia due to drugs and toxins |
| **ASTHMA** | |
| **Included ICD-10 codes and labels** | |
| J45 | Asthma |
| **ATRIAL FIBRILLATION** | |
| **Included ICD-10 codes and labels** | |
| I48 | Atrial fibrillation and flutter |
| **AUTOIMMUNE DISEASES** | |
| **Included ICD-10 codes and labels** | |
| I731 | Thromboangiitis obliterans [Buerger] |
| L10 | Pemphigus |
| L12 | Pemphigoid |
| L40 | Psoriasis |
| L41 | Parapsoriasis |
| L93 | Lupus erythematosus |
| L94 | Other localized connective tissue disorders |
| L95 | Vasculitis limited to skin, not elsewhere classified |
| M30 | Polyarteritis nodosa and related conditions |
| M31 | Other necrotizing vasculopathies |
| M32 | Systemic lupus erythematosus |
| M33 | Dermatopolymyositis |
| M34 | Systemic sclerosis |
| M35 | Other systemic involvement of connective tissue |
| M36 | Systemic disorders of connective tissue in diseases classified elsewhere |
| **Excluded ICD-10 codes and labels** | |
| L105 | Drug-induced pemphigus |
| M320 | Drug-induced systemic lupus erythematosus |
| M342 | Systemic sclerosis induced by drugs and chemicals |
| M357 | Hypermobility syndrome |
| M358 | Other specified systemic involvement of connective tissue |
| M359 | Systemic involvement of connective tissue, unspecified |
| M360 | Dermato(poly)myositis in neoplastic disease |
| M361 | Arthropathy in neoplastic disease |
| M362 | Haemophilic arthropathy |
| M363 | Arthropathy in other blood disorders |
| **BLINDNESS, VISUAL IMPAIRMENT** | |
| **Included ICD-10 codes and labels** | |
| H54 | Visual impairment including blindness (binocular or monocular) |
| Z442 | Fitting and adjustment of artificial eye |
| Z970 | Presence of artificial eye |
| **Excluded ICD-10 codes and labels** | |
| H543 | Mild or no visual impairment, binocular |
| **BLOOD AND BLOOD FORMING ORGAN DISEASES** | |
| **Included ICD-10 codes and labels** | |
| D66 | Hereditary factor VIII deficiency |
| D67 | Hereditary factor IX deficiency |
| D68 | Other coagulation defects |
| D69 | Purpura and other haemorrhagic conditions |
| D71 | Functional disorders of polymorphonuclear neutrophils |
| D720 | Genetic anomalies of leukocytes |
| D730 | Hyposplenism |
| D731 | Hypersplenism |
| D732 | Chronic congestive splenomegaly |
| D74 | Methaemoglobinaemia |
| D750 | Familial erythrocytosis |
| D761 | Haemophagocytic lymphohistiocytosis |
| D763 | Other histiocytosis syndromes |
| D77 | Other disorders of blood and blood-forming organs in diseases classified elsewhere |
| D80 | Immunodeficiency with predominantly antibody defects |
| D81 | Combined immunodeficiencies |
| D82 | Immunodeficiency associated with other major defects |
| D83 | Common variable immunodeficiency |
| D84 | Other immunodeficiencies |
| D86 | Sarcoidosis |
| D89 | Other disorders involving the immune mechanism, not elsewhere classified |
| **Excluded ICD-10 codes and labels** | |
| D683 | Haemorrhagic disorder due to circulating anticoagulants |
| D684 | Acquired coagulation factor deficiency |
| D695 | Secondary thrombocytopenia |
| D748 | Other methaemoglobinaemias |
| D807 | Transient hypogammaglobulinaemia of infancy |
| D891 | Cryoglobulinaemia |
| D893 | Immune reconstitution syndrome |
| **BRADYCARDIAS AND CONDUCTION DISEASES** | |
| **Included ICD-10 codes and labels** | |
| I441 | Atrioventricular block, second degree |
| I442 | Atrioventricular block, complete |
| I443 | Other and unspecified atrioventricular block |
| I453 | Trifascicular block |
| I455 | Other specified heart block |
| Z950 | Presence of cardiac pacemaker |
| **CARDIAC VALVE DISEASES** | |
| **Included ICD-10 codes and labels** | |
| I05 | Rheumatic mitral valve diseases |
| I06 | Rheumatic aortic valve diseases |
| I07 | Rheumatic tricuspid valve diseases |
| I08 | Multiple valve diseases |
| I091 | Rheumatic diseases of endocardium, valve unspecified |
| I098 | Other specified rheumatic heart diseases |
| I34 | Nonrheumatic mitral valve disorders |
| I35 | Nonrheumatic aortic valve disorders |
| I36 | Nonrheumatic tricuspid valve disorders |
| I37 | Pulmonary valve disorders |
| I38 | Endocarditis, valve unspecified |
| I390 | Mitral valve disorders in diseases classified elsewhere |
| I391 | Aortic valve disorders in diseases classified elsewhere |
| I392 | Tricuspid valve disorders in diseases classified elsewhere |
| I393 | Pulmonary valve disorders in diseases classified elsewhere |
| I394 | Multiple valve disorders in diseases classified elsewhere |
| Q22 | Congenital malformations of pulmonary and tricuspid valves |
| Q23 | Congenital malformations of aortic and mitral valves |
| Z952 | Presence of prosthetic heart valve |
| Z953 | Presence of xenogenic heart valve |
| Z954 | Presence of other heart-valve replacement |
| **CATARACT AND OTHER LENS DISEASES** | |
| **Included ICD-10 codes and labels** | |
| H25 | Senile cataract |
| H26 | Other cataract |
| H27 | Other disorders of lens |
| H28 | Cataract and other disorders of lens in diseases classified elsewhere |
| Q12 | Congenital lens malformations |
| Z961 | Presence of intraocular lens |
| **CEREBROVASCULAR DISEASE** | |
| **Included ICD-10 codes and labels** | |
| G45 | Transient cerebral ischaemic attacks and related syndromes |
| G46 | Vascular syndromes of brain in cerebrovascular diseases |
| I60 | Subarchnoid hemorrhage |
| I61 | Intracerebral haemorrhage |
| I62 | Other nontraumatic intracranial haemorrhage |
| I63 | Cerebral infarction |
| I64 | Stroke, not specified as haemorrhage or infarction |
| I67 | Other cerebrovascular diseases |
| I69 | Sequelae of cerebrovascular disease |
| **CHROMOSOMAL ABNORMALITIES** | |
| **Included ICD-10 codes and labels** | |
| Q90 | Down syndrome |
| Q91 | Edwards syndrome and Patau syndrome |
| Q92 | Other trisomies and partial trisomies of the autosomes, not elsewhere classified |
| Q93 | Monosomies and deletions from the autosomes, not elsewhere classified |
| Q95 | Balanced rearrangements and structural markers, not elsewhere classified |
| Q96 | Turner syndrome |
| Q97 | Other sex chromosome abnormalities, female phenotype, not elsewhere classified |
| Q98 | Other sex chromosome abnormalities, male phenotype, not elsewhere classified |
| Q99 | Other chromosome abnormalities, not elsewhere classified |
| **CHRONIC INFECTIOUS DISEASES** | |
| **Included ICD-10 codes and labels** | |
| A15 | Respiratory tuberculosis, bacteriologically and histologically confirmed |
| A16 | Respiratory tuberculosis, not confirmed bacteriologically or histologically |
| A17 | Tuberculosis of nervous system |
| A18 | Tuberculosis of other organs |
| A19 | Miliary tuberculosis |
| A30 | Leprosy [Hansen disease] |
| A31 | Infection due to other mycobacteria |
| A50 | Congenital syphilis |
| A52 | Late syphilis |
| A53 | Other and unspecified syphilis |
| A65 | Nonvenereal syphilis |
| A66 | Yaws |
| A67 | Pint [carat] |
| A692 | Lyme disease |
| A81 | Atypical virus infections of central nervous system |
| B20 | Human immunodeficiency virus [HIV] disease resulting in infectious and parasitic diseases |
| B21 | Human immunodeficiency virus [HIV] disease resulting in malignant neoplasms |
| B22 | Human immunodeficiency virus [HIV] disease resulting in other specified diseases |
| B23 | Human immunodeficiency virus [HIV] disease resulting in other conditions |
| B24 | Unspecified human immunodeficiency virus [HIV] disease |
| B381 | Chronic pulmonary coccidioidomycosis |
| B391 | Chronic pulmonary histoplasmosis capsulati |
| B401 | Chronic pulmonary blastomycosis |
| B572 | Chagas disease (chronic) with heart involvement |
| B573 | Chagas disease (chronic) with digestive system involvement |
| B574 | Chagas disease (chronic) with nervous system involvement |
| B575 | Chagas disease (chronic) with other organ involvement |
| B65 | Schistosomiasis [bilharziasis] |
| B92 | Sequelae or leprosy |
| B94 | Sequelae of other and unspecified infectious and parasitic diseases |
| J65 | Pneumoconiosis associated with tuberculosis |
| M863 | Chronic multifocal osteomyelitis |
| M864 | Chronic osteomyelitis with draining sinus |
| M865 | Other chronic haematogenous osteomyelitis |
| M866 | Other chronic osteomyelitis |
| **CHRONIC KIDNEY DISEASES** | |
| **Included ICD-10 codes and labels** | |
| I120 | Hypertensive renal disease with renal failure |
| I130 | Hypertensive heart and renal disease with (congestive) heart failure |
| I131 | Hypertensive heart and renal disease with renal failure |
| I132 | Hypertensive heart and renal disease with both (congestive) heart failure and renal failure |
| I139 | Hypertensive heart and renal disease, unspecified |
| N01 | Rapidly progressive nephritic syndrome |
| N03 | Chronic nephritic syndrome |
| N04 | Nephrotic syndrome |
| N05 | Unspecified nephritic syndrome |
| N07 | Hereditary nephropathy, not elsewhere classified |
| N08 | Glomerular disorders in diseases classified elsewhere |
| N11 | Chronic tubulo-interstitial nephritis |
| N183 | Chronic kidney disease, stage 3 |
| N184 | Chronic kidney disease, stage 4 |
| N185 | Chronic kidney disease, stage 5 |
| N189 | Chronic kidney disease, unspecified |
| Q60 | Renal agenesis and other reduction defects of kidney |
| Q611 | Polycystic kidney, autosomal recessive |
| Q612 | Polycystic kidney, autosomal dominant |
| Q613 | Polycystic kidney, unspecified |
| Q614 | Renal dysplasia |
| Q615 | Medullary cystic kidney |
| Q618 | Other cystic kidney diseases |
| Q619 | Cystic kidney disease, unspecified |
| Z905 | Acquired absence of kidney |
| Z940 | Kidney transplant status |
| **CHRONIC LIVER DISEASES** | |
| **Included ICD-10 codes and labels** | |
| B18 | Chronic viral hepatitis |
| K70 | Alcoholic liver disease |
| K713 | Toxic liver disease with chronic persistent hepatitis |
| K714 | Toxic liver disease with chronic lobular hepatitis |
| K715 | Toxic liver disease with chronic active hepatitis |
| K717 | Toxic liver disease with fibrosis and cirrhosis of liver |
| K721 | Chronic hepatic failure |
| K73 | Chronic hepatitis, not elsewhere classified |
| K74 | Fibrosis and cirrhosis of liver |
| K753 | Granulomatous hepatitis, not elsewhere classified |
| K754 | Autoimmune hepatitis |
| K758 | Other specified inflammatory liver diseases |
| K761 | Chronic passive congestion of liver |
| K766 | Portal hypertension |
| K767 | Hepatorenal syndrome |
| K778 | Liver disorders in other diseases classified elsewhere |
| Q446 | Cystic disease of liver |
| Z944 | Liver transplant status |
| **Excluded ICD-10 codes and labels** | |
| K700 | Alcoholic fatty liver |
| K701 | Alcoholic hepatitis |
| **CHRONIC PANCREAS, BILIARY TRACT AND GALLBLADDER DISEASES** | |
| **Included ICD-10 codes and labels** | |
| K800 | Calculus of gallbladder with acute cholecystitis |
| K801 | Calculus of gallbladder with other cholecystitis |
| K802 | Calculus of gallbladder without cholecystitis |
| K808 | Other cholelithiasis |
| K811 | Chronic cholecystitis |
| K86 | Other diseases of pancreas |
| Q440 | Agenesis, aplasia and hypoplasia of gallbladder |
| Q441 | Other congenital malformations of gallbladder |
| Q442 | Atresia of bile ducts |
| Q443 | Congenital stenosis and stricture of bile ducts |
| Q444 | Choledochal cyst |
| Q445 | Other congenital malformations of bile ducts |
| Q450 | Agenesis, aplasia and hypoplasia of pancreas |
| **Excluded ICD-10 codes and labels** | |
| K862 | Cyst of pancreas |
| K863 | Pseudocyst of pancreas |
| K869 | Disease of pancreas, unspecified |
| **CHRONIC ULCER OF THE SKIN** | |
| **Included ICD-10 codes and labels** | |
| I830 | Varicose veins of lower extremities with ulcer |
| I832 | Varicose veins of lower extremities with both ulcer and inflammation |
| L89 | Decubitus ulcer and pressure area |
| L97 | Ulcer of lower limb, not elsewhere classified |
| L984 | Chronic ulcer of skin, not elsewhere classified |
| **COLITIS AND RELATED DISEASES** | |
| **Included ICD-10 codes and labels** | |
| K520 | Gastroenteritis and colitis due to radiation |
| K528 | Other specified noninfective gastroenteritis and colitis |
| K551 | Chronic vascular disorders of intestine |
| K552 | Angiodysplasia of colon |
| K572 | Diverticular disease of large intestine with perforation and abscess |
| K573 | Diverticular disease of large intestine without perforation or abscess |
| K574 | Diverticular disease of both small and large intestine with perforation and abscess |
| K575 | Diverticular disease of both small and large intestine without perforation or abscess |
| K578 | Diverticular disease of intestine, part unspecified, with perforation and abscess |
| K579 | Diverticular disease of intestine, part unspecified, without perforation or abscess |
| K58 | Irritable bowel syndrome |
| K590 | Constipation |
| K592 | Neurogenic bowel, not elsewhere classified |
| K62 | Other diseases of anus and rectum |
| K634 | Enteroptosis |
| K64 | Haemorrhoids and perianal venous thrombosis |
| **Excluded ICD-10 codes and labels** | |
| K620 | Anal polyp |
| K621 | Rectal polyp |
| K625 | Haemorrhage of anus and rectum |
| K626 | Ulcer of anus and rectum |
| K645 | Perianal venous thrombosis |
| **COPD, EMPHYSEMA, CHRONIC BRONCHITIS** | |
| **Included ICD-10 codes and labels** | |
| J41 | Simple and mucopurulent chronic bronchitis |
| J42 | Unspecified chronic bronchitis |
| J43 | Emphysema |
| J44 | Other chronic obstructive pulmonary disease |
| J47 | Bronchiectasis |
| **DEAFNESS, HEARING IMPAIRMENT** | |
| **Included ICD-10 codes and labels** | |
| H80 | Otosclerosis |
| H90 | Conductive and sensorineural hearing loss |
| H911 | Presbycusis |
| H913 | Deaf mutism, not elsewhere classified |
| H919 | Hearing loss, unspecified |
| Q16 | Congenital malformations of ear causing impairment of hearing |
| Z453 | Adjustment and management of implanted hearing device |
| Z461 | Fitting and adjustment of hearing aid |
| Z962 | Presence of otological and audiological implants |
| Z974 | Presence of external hearing-aid |
| **DEMENTIA** | |
| **Included ICD-10 codes and labels** | |
| F00 | Dementia in Alzheimer disease |
| F01 | Vascular dementia |
| F02 | Dementia in other diseases classified elsewhere |
| F03 | Unspecified dementia |
| F051 | Delirium superimposed on dementia |
| G30 | Alzheimer disease |
| G31 | Other degenerative diseases of nervous system, not elsewhere classified |
| **DEPRESSION AND MOOD DISEASES** | |
| **Included ICD-10 codes and labels** | |
| F30 | Manic episode |
| F31 | Bipolar affective disorder |
| F32 | Depressive episode |
| F33 | Recurrent depressive disorder |
| F34 | Persistent mood [affective] disorders |
| F38 | Other mood [affective] disorders |
| F39 | Unspecified mood [affective] disorder |
| F412 | Mixed anxiety and depressive disorder |
| **DIABETES** | |
| **Included ICD-10 codes and labels** | |
| E10 | Insulin-dependent diabetes mellitus |
| E11 | Non-insulin-dependent diabetes mellitus |
| E13 | Other specified diabetes mellitus |
| E14 | Unspecified diabetes mellitus |
| E891 | Postprocedural hypoinsulinaemia |
| **DORSOPATHIES** | |
| **Included ICD-10 codes and labels** | |
| M40 | Kyphosis and lordosis |
| M41 | Scoliosis |
| M42 | Spinal osteochondrosis |
| M43 | Other deforming dorsopathies |
| M47 | Spondylosis |
| M48 | Other spondylopathies |
| M49 | Spondylopathies in diseases classified elsewhere |
| M50 | Cervical disc disorders |
| M51 | Other intervertebral disc disorders |
| M53 | Other dorsopathies, not elsewhere classified |
| Q675 | Congenital deformity of spine |
| Q761 | Klippel-Feil syndrome |
| Q764 | Other congenital malformations of spine, not associated with scoliosis |
| **DYSLIPIDEMIA** | |
| **Included ICD-10 codes and labels** | |
| E78 | Disorders of lipoprotein metabolism and other lipidaemias |
| **EAR, NOSE, THROAT DISEASES** | |
| **Included ICD-10 codes and labels** | |
| H604 | Cholesteatoma of external ear |
| H661 | Chronic tubotympanic suppurative otitis media |
| H662 | Chronic atticoantral suppurative otitis media |
| H663 | Other chronic suppurative otitis media |
| H701 | Chronic mastoiditis |
| H71 | Cholesteatoma of middle ear |
| H731 | Chronic myringitis |
| H741 | Adhesive middle ear disease |
| H810 | The disease |
| H831 | Labyrinthine fistula |
| H832 | Labyrinthine dysfunction |
| H95 | Postprocedural disorders of ear and mastoid process, not elsewhere classified |
| J300 | Vasomotor rhinitis |
| J31 | Chronic rhinitis, nasopharyngitis and pharyngitis |
| J32 | Chronic sinusitis |
| J33 | Nasal polyp |
| J341 | Cyst and mucocele of nose and nasal sinus |
| J342 | Deviated nasal septum |
| J343 | Hypertrophy of nasal turbinates |
| J35 | Chronic diseases of tonsils and adenoids |
| J37 | Chronic laryngitis and laryngotracheitis |
| J380 | Paralysis of vocal cords and larynx |
| J386 | Stenosis of larynx |
| K051 | Chronic gingivitis |
| K053 | Chronic periodontitis |
| K07 | Dentofacial anomalies [including malocclusion] |
| K110 | Atrophy of salivary gland |
| K117 | Disturbances of salivary secretion |
| Q30 | Congenital malformations of nose |
| Q31 | Congenital malformations of larynx |
| Q32 | Congenital malformations of trachea and bronchus |
| Q35 | Cleft palate |
| Q36 | Cleft lip |
| Q37 | Cleft palate with cleft lip |
| Q38 | Other congenital malformations of tongue, mouth and pharynx |
| **EPILEPSY** | |
| **Included ICD-10 codes and labels** | |
| G40 | Epilepsy |
| **Excluded ICD-10 codes and labels** | |
| G405 | Special epileptic syndromes |
| **ESOPHAGUS, STOMACH AND DUODENUM DISEASES** | |
| **Included ICD-10 codes and labels** | |
| I85 | Osophageal varicoses |
| I864 | Gastric varices |
| I982 | Oesophageal varices without bleeding in diseases classified elsewhere |
| I983 | Oesophageal varices with bleeding in diseases classified elsewhere |
| K21 | Gastro-oesophageal reflux disease |
| K220 | Achalasia of cardia |
| K222 | Oesophageal obstruction |
| K224 | Dyskinesia of oesophagus |
| K225 | Diverticulum of oesophagus, acquired |
| K227 | Barrett oesophagus |
| K230 | Tuberculous oesophagitis |
| K231 | Megaoesophagus in Chagas disease |
| K254 | Gastric ulcer: Chronic or unspecified with haemorrhage |
| K255 | Gastric ulcer: Chronic or unspecified with perforation |
| K256 | Gastric ulcer: Chronic or unspecified with both haemorrhage and perforation |
| K257 | Gastric ulcer: Chronic without haemorrhage or perforation |
| K264 | Duodenal ulcer: Chronic or unspecified with haemorrhage |
| K265 | Duodenal ulcer: Chronic or unspecified with perforation |
| K266 | Duodenal ulcer: Chronic or unspecified with both haemorrhage and perforation |
| K267 | Duodenal ulcer: Chronic without haemorrhage or perforation |
| K274 | Peptic ulcer, site unspecified: Chronic or unspecified with haemorrhage |
| K275 | Peptic ulcer, site unspecified: Chronic or unspecified with perforation |
| K276 | Peptic ulcer, site unspecified: Chronic or unspecified with both haemorrhage and perforation |
| K277 | Peptic ulcer, site unspecified: Chronic without haemorrhage or perforation |
| K284 | Gastrojejunal ulcer: Chronic or unspecified with haemorrhage |
| K285 | Gastrojejunal ulcer: Chronic or unspecified with perforation |
| K286 | Gastrojejunal ulcer: Chronic or unspecified with both haemorrhage and perforation |
| K287 | Gastrojejunal ulcer: Chronic without haemorrhage or perforation |
| K293 | Chronic superficial gastritis |
| K294 | Chronic atrophic gastritis |
| K295 | Chronic gastritis, unspecified |
| K296 | Other gastritis |
| K297 | Gastritis, unspecified |
| K298 | Duodenitis |
| K299 | Gastroduodenitis, unspecified |
| K311 | Adult hypertrophic pyloric stenosis |
| K312 | Hourglass stricture and stenosis of stomach |
| K313 | Pylorospasm, not elsewhere classified |
| K314 | Gastric diverticulum |
| K315 | Abstraction of Duodenuma |
| Q39 | Congenital malformations of oesophagus |
| Q40 | Other congenital malformations of upper alimentary tract |
| Z903 | Acquired absence of part of stomach |
| **GLAUCOMA** | |
| **Included ICD-10 codes and labels** | |
| H401 | Primary open-angle glaucoma |
| H402 | Primary angle-closure glaucoma |
| H403 | Glaucoma secondary to eye trauma |
| H404 | Glaucoma secondary to eye inflammation |
| H405 | Glaucoma secondary to other eye disorders |
| H406 | Glaucoma secondary to drugs |
| H408 | Other glaucoma |
| H409 | Glaucoma, unspecified |
| **HEART FAILURE** | |
| **Included ICD-10 codes and labels** | |
| I110 | Hypertensive heart disease with (congestive) heart failure |
| I130 | Hypertensive heart and renal disease with (congestive) heart failure |
| I132 | Hypertensive heart and renal disease with both (congestive) heart failure and renal failure |
| I27 | Other pulmonary heart diseases |
| I280 | Arteriovenous fistula of pulmonary vessels |
| I42 | Cardiomyopathy |
| I43 | Cardiomyopathy in diseases classified elsewhere |
| I50 | Heart failure |
| I515 | Myocardial degeneration |
| I517 | Cardiomegaly |
| I528 | Other heart disorders in other diseases classified elsewhere |
| Z941 | Heart transplant status |
| Z943 | Heart and lungs transplant status |
| **HEMATOLOGICAL NEOPLASMS** | |
| **Included ICD-10 codes and labels** | |
| C81 | Hodgkin lymphoma |
| C82 | Follicular lymphoma |
| C83 | Non-follicular lymphoma |
| C84 | Mature T/NK-cell lymphomas |
| C85 | Other and unspecified types of non-Hodgkin lymphoma |
| C86 | Other specified types of T/NK-cell lymphoma |
| C88 | Malignant immunoproliferative diseases |
| C90 | Multiple myeloma and malignant plasma cell neoplasms |
| C91 | Lymphoid leukaemia |
| C92 | Myeloid leukaemia |
| C93 | Monocytic leukaemia |
| C94 | Other leukaemias of specified cell type |
| C95 | Leukaemia of unspecified cell type |
| C96 | Other and unspecified malignant neoplasms of lymphoid, haematopoietic and related tissue |
| **HYPERTENSION** | |
| **Included ICD-10 codes and labels** | |
| I10 | Essential (primary) hypertension |
| I11 | Hypertensive heart disease |
| I12 | Hypertensive renal disease |
| I13 | Hypertensive heart and renal disease |
| I15 | Secondary hypertension |
| **INFLAMMATORY ARTHROPATHIES** | |
| **Included ICD-10 codes and labels** | |
| M023 | Reiter disease |
| M05 | Seropositive rheumatoid arthritis |
| M06 | Other rheumatoid arthritis |
| M07 | Psoriatic and enteropathic arthropathies |
| M08 | Juvenile arthritis |
| M09 | Juvenile arthritis in diseases classified elsewhere |
| M10 | Taste |
| M11 | Other crystal arthropathies |
| M12 | Other specific arthropathies |
| M13 | Other arthritis |
| M14 | Arthropathies in other diseases classified elsewhere |
| M45 | Ankylosing spondylitis |
| M460 | Spinal enthesopathy |
| M461 | Sacroiliitis, not elsewhere classified |
| M468 | Other specified inflammatory spondylopathies |
| M469 | Inflammatory spondylopathy, unspecified |
| **INFLAMMATORY BOWEL DISEASES** | |
| **Included ICD-10 codes and labels** | |
| K50 | Crohn disease [regional enteritis] |
| K51 | Ulcerative colitis |
| **ISCHEMIC HEART DISEASE** | |
| **Included ICD-10 codes and labels** | |
| I20 | Angina pectoris |
| I21 | Acute myocardial infarction |
| I22 | Subsequent myocardial infarction |
| I24 | Other acute ischaemic heart diseases |
| I25 | Chronic ischaemic heart disease |
| Z951 | Presence of aortocoronary bypass graft |
| Z955 | Presence of coronary angioplasty implant and graft |
| **MIGRAINE AND FACIAL PAIN SYNDROMES** | |
| **Included ICD-10 codes and labels** | |
| G43 | Migraine |
| G440 | Cluster headache syndrome |
| G441 | Vascular headache, not elsewhere classified |
| G442 | Tension-type headache |
| G443 | Chronic post-traumatic headache |
| G448 | Other specified headache syndromes |
| G50 | Disorders of trigeminal nerve |
| **MULTIPLE SCLEROSIS** | |
| **Included ICD-10 codes and labels** | |
| G35 | Multiple sclerosis |
| **NEUROTIC, STRESS-RELATED AND SOMATOFORM DISEASES** | |
| **Included ICD-10 codes and labels** | |
| F40 | Phobic anxiety disorders |
| F41 | Other anxiety disorders |
| F42 | Obsessive-compulsive disorder |
| F43 | Reaction to severe stress, and adjustment disorders |
| F44 | Dissociative [conversion] disorders |
| F45 | Somatoform disorders |
| F48 | Other neurotic disorders |
| **Excluded ICD-10 codes and labels** | |
| F430 | Acute stress reaction |
| F432 | Adjustment disorders |
| **OBESITY** | |
| **Included ICD-10 codes and labels** | |
| E66 | Obesity |
| **OSTEOARTHRITIS AND OTHER DEGENERATIVE JOINT DISEASES** | |
| **Included ICD-10 codes and labels** | |
| M15 | Polyarthrosis |
| M16 | Coxarthrosis [arthrosis of hip] |
| M17 | Gonarthrosis [arthrosis of knee] |
| M18 | Arthrosis of first carpometacarpal joint |
| M19 | Other arthrosis |
| M362 | Haemophilic arthropathy |
| M363 | Arthropathy in other blood disorders |
| **OSTEOPOROSIS** | |
| **Included ICD-10 codes and labels** | |
| M80 | Osteoporosis with pathological fracture |
| M81 | Osteoporosis without pathological fracture |
| M82 | Osteoporosis in diseases classified elsewhere |
| **OTHER CARDIOVASCULAR DISEASES** | |
| **Included ICD-10 codes and labels** | |
| I09 | Other rheumatic heart diseases |
| I281 | Aneurysm of pulmonary artery |
| I310 | Chronic adhesive pericarditis |
| I311 | Chronic constrictive pericarditis |
| I456 | Pre-excitation syndrome |
| I495 | Sick sinus syndrome |
| I498 | Other specified cardiac arrhythmias |
| I70 | Atherosclerosis |
| I71 | Aortic aneurysm and dissection |
| I72 | Other aneurysm and dissection |
| I790 | Aneurysm of aorta in diseases classified elsewhere |
| I791 | Aortitis in diseases classified elsewhere |
| I950 | Idiopathic hypotension |
| I951 | Orthostatic hypotension |
| I958 | Other hypotension |
| Q20 | Congenital malformations of cardiac chambers and connections |
| Q21 | Congenital malformations of cardiac septa |
| Q24 | Other congenital malformations of heart |
| Q25 | Congenital malformations of great arteries |
| Q26 | Congenital malformations of great veins |
| Q27 | Other congenital malformations of peripheral vascular system |
| Q28 | Other congenital malformations of circulatory system |
| Z958 | Presence of other cardiac and vascular implants and grafts |
| Z959 | Presence of cardiac and vascular implant and graft, unspecified |
| **Excluded ICD-10 codes and labels** | |
| I091 | Rheumatic diseases of endocardium, valve unspecified |
| I098 | Other specified rheumatic heart diseases |
| I702 | Atherosclerosis of arteries of extremities |
| **OTHER DIGESTIVE DISEASES** | |
| **Included ICD-10 codes and labels** | |
| K660 | Peritoneal adhesions |
| K900 | Coeliac disease |
| K901 | Tropical sprue |
| K902 | Blind loop syndrome, not elsewhere classified |
| K911 | Postgastric surgery syndromes |
| K93 | Disorders of other digestive organs in diseases classified elsewhere |
| Q41 | Congenital absence, atresia and stenosis of small intestine |
| Q42 | Congenital absence, atresia and stenosis of large intestine |
| Q43 | Other congenital malformations of intestine |
| R15 | Faecal incontinence |
| Z904 | Acquired absence of other parts of digestive tract |
| Z980 | Intestinal bypass and anastomosis status |
| **OTHER EYE DISEASES** | |
| **Included ICD-10 codes and labels** | |
| H022 | Lagophthalmos |
| H023 | Blepharochalasis |
| H024 | Ptosis of eyelid |
| H025 | Other disorders affecting eyelid function |
| H04 | Disorders of lacrimal system |
| H05 | Disorders of orbit |
| H104 | Chronic conjunctivitis |
| H17 | Corneal scars and opacities |
| H184 | Corneal degeneration |
| H185 | Hereditary corneal dystrophies |
| H186 | Keratoconus |
| H187 | Other corneal deformities |
| H188 | Other specified disorders of cornea |
| H189 | Disorder of cornea, unspecified |
| H193 | Keratitis and keratoconjunctivitis in other diseases classified elsewhere |
| H198 | Other disorders of sclera and cornea in diseases classified elsewhere |
| H201 | Chronic iridocyclitis |
| H21 | Other disorders of iris and ciliary body |
| H310 | Chorioretinal scars |
| H311 | Charydal Degeneration |
| H312 | Hereditary choroidal dystrophy |
| H318 | Other specified disorders of choroid |
| H319 | Disorder of choroid, unspecified |
| H33 | Retinal detachments and breaks |
| H352 | Other proliferative retinopathy |
| H353 | Degeneration of macula and posterior pole |
| H354 | Peripheral retinal degeneration |
| H355 | Hereditary retinal dystrophy |
| H357 | Separation of retinal layers |
| H358 | Other specified retinal disorders |
| H359 | Retinal disorder, unspecified |
| H36 | Retinal disorders in diseases classified elsewhere |
| H47 | Other disorders of optic [2nd] nerve and visual pathways |
| H48 | Disorders of optic [2nd] nerve and visual pathways in diseases classified elsewhere |
| H49 | Paralytic strabismus |
| H51 | Other disorders of binocular movement |
| Q10 | Congenital malformations of eyelid, lacrimal apparatus and orbit |
| Q11 | Anophthalmos, microphthalmos and macrophthalmos |
| Q13 | Congenital malformations of anterior segment of eye |
| Q14 | Congenital malformations of posterior segment of eye |
| Q15 | Other congenital malformations of eye |
| Z947 | Corneal transplant status |
| **Excluded ICD-10 codes and labels** | |
| H043 | Acute and unspecified inflammation of lacrimal passages |
| H050 | Acute inflammation of orbit |
| H470 | Disorders of optic nerve, not elsewhere classified |
| H471 | Papilloedema, unspecified |
| H481 | Retrobulbar neuritis in diseases classified elsewhere |
| **OTHER GENITOURINARY DISEASES** | |
| **Included ICD-10 codes and labels** | |
| B901 | Sequelae of genitourinary tuberculosis |
| N200 | Calculus of kidney |
| N202 | Calculus of kidney with calculus of ureter |
| N209 | Urinary calculus, unspecified |
| N210 | Calculus in bladder |
| N218 | Other lower urinary tract calculus |
| N219 | Calculus of lower urinary tract, unspecified |
| N22 | Calculus of urinary tract in diseases classified elsewhere |
| N301 | Interstitial cystitis (chronic) |
| N302 | Other chronic cystitis |
| N303 | Trigonitis |
| N304 | Irradiation cystitis |
| N31 | Neuromuscular dysfunction of bladder, not elsewhere classified |
| N320 | Bladder-neck obstruction |
| N323 | Diverticulum of bladder |
| N328 | Other specified disorders of bladder |
| N329 | Bladder disorder, unspecified |
| N33 | Bladder disorders in diseases classified elsewhere |
| N35 | Urethral stricture |
| N393 | Stress incontinence |
| N394 | Other specified urinary incontinence |
| N480 | Leukoplakia or penis |
| N484 | Impotence of organic origin |
| N489 | Disorder of penis, unspecified |
| N701 | Chronic salpingitis and oophoritis |
| N711 | Chronic inflammatory disease of uterus |
| N731 | Chronic parametritis and pelvic cellulitis |
| N734 | Female chronic pelvic peritonitis |
| N736 | Female pelvic peritoneal adhesions |
| N761 | Subacute and chronic vaginitis |
| N763 | Subacute and chronic vulvitis |
| N81 | Female genital prolapse |
| N88 | Other noninflammatory disorders of cervix uteri |
| N895 | Stricture and atresia of vagina |
| N905 | Atrophy of vulva |
| N952 | Postmenopausal atrophic vaginitis |
| Q54 | Hypospadias |
| Q620 | Congenital hydronephrosis |
| Q621 | Atresia and stenosis of ureter |
| Q622 | Congenital megaloureter |
| Q623 | Other obstructive defects of renal pelvis and ureter |
| Q624 | Agenesis of ureter |
| Q627 | Congenital vesico-uretero-renal reflux |
| Q628 | Other congenital malformations of ureter |
| Q638 | Other specified congenital malformations of kidney |
| Q639 | Congenital malformation of kidney, unspecified |
| Q640 | Eepispadias |
| Q641 | Exstrophy of urinary bladder |
| Q643 | Other atresia and stenosis of urethra and bladder neck |
| Q644 | Malformation of urachus |
| Q645 | Congenital absence of bladder and urethra |
| Q646 | Congenital diverticulum of bladder |
| Q647 | Other congenital malformations of bladder and urethra |
| Q648 | Other specified congenital malformations of urinary system |
| Q649 | Congenital malformation of urinary system, unspecified |
| Z906 | Acquired absence of other organs of urinary tract |
| Z907 | Acquired absence of genital organ(s) |
| Z960 | Presence of urogenital implants |
| **OTHER METABOLIC DISEASES** | |
| **Included ICD-10 codes and labels** | |
| E20 | Hypoparathyroidism |
| E21 | Hyperparathyroidism and other disorders of parathyroid gland |
| E22 | Hyperfunction of pituitary gland |
| E23 | Hypofunction and other disorders of pituitary gland |
| E24 | Cushing syndrome |
| E25 | Adrenogenital disorders |
| E26 | Hyperaldosteronism |
| E27 | Other disorders of adrenal gland |
| E28 | Ovarian dysfunction |
| E29 | Testicular dysfunction |
| E31 | Polyglandular dysfunction |
| E34 | Other endocrine disorders |
| E35 | Disorders of endocrine glands in diseases classified elsewhere |
| E40 | Kwashiorkor |
| E41 | Nutritional marasmus |
| E42 | Marasmic kwashiorkor |
| E43 | Unspecified severe protein-energy malnutrition |
| E44 | Protein-energy malnutrition of moderate and mild degree |
| E45 | Retarded development following protein-energy malnutrition |
| E46 | Unspecified protein-energy malnutrition |
| E64 | Sequelae of malnutrition and other nutritional deficiencies |
| E70 | Disorders of aromatic amino-acid metabolism |
| E71 | Disorders of branched-chain amino-acid metabolism and fatty-acid metabolism |
| E72 | Other disorders of amino-acid metabolism |
| E74 | Other disorders of carbohydrate metabolism |
| E75 | Disorders of sphingolipid metabolism and other lipid storage disorders |
| E76 | Disorders of glycosaminoglycan metabolism |
| E77 | Disorders of glycoprotein metabolism |
| E79 | Disorders of purine and pyrimidine metabolism |
| E80 | Disorders of porphyrin and bilirubin metabolism |
| E83 | Disorders of mineral metabolism |
| E84 | Cystic fibrosis |
| E85 | Amyloidosis |
| E88 | Other metabolic disorders |
| E89 | Postprocedural endocrine and metabolic disorders, not elsewhere classified |
| K903 | Pancreatic steatorrhoea |
| K904 | Malabsorption due to intolerance, not elsewhere classified |
| K908 | Other intestinal malabsorption |
| K909 | Intestinal malabsorption, unspecified |
| K912 | Postsurgical malabsorption, not elsewhere classified |
| M83 | Adult osteomalacia |
| M88 | Paget disease of bone [osteitis deformans] |
| N25 | Disorders resulting from impaired renal tubular function |
| **Excluded ICD-10 codes and labels** | |
| E231 | Drug-induced hypopituitarism |
| E242 | Drug-induced Cushing syndrome |
| E244 | Alcohol-induced pseudo-Cushing syndrome |
| E273 | Drug-induced adrenocortical insufficiency |
| E343 | Short stature, not elsewhere classified |
| E344 | Constitutional tall stature |
| E350 | Disorders of thyroid gland in diseases classified elsewhere |
| E441 | Mild protein-energy malnutrition |
| E790 | Hyperuricaemia without signs of inflammatory arthritis and tophaceous disease |
| E804 | Gilbert syndrome |
| E883 | Tumour lysis syndrome |
| E890 | Postprocedural hypothyroidism |
| E892 | Postprocedural hypoparathyroidism |
| **OTHER MUSCULOSKELETAL AND JOINT DISEASES** | |
| **Included ICD-10 codes and labels** | |
| B902 | Sequelae of tuberculosis of bones and joints |
| M212 | Flexion deformity |
| M213 | Wrist or foot drop (acquired) |
| M214 | Flat foot [pes planus] (acquired) |
| M215 | Acquired clawhand, clubhand, clawfoot and clubfoot |
| M216 | Other acquired deformities of ankle and foot |
| M217 | Unequal limb length (acquired) |
| M218 | Other specified acquired deformities of limbs |
| M219 | Acquired deformity of limb, unspecified |
| M22 | Disorders of patella |
| M23 | Internal derangement of knee |
| M24 | Other specific joint derangements |
| M252 | Flail joint |
| M253 | Other instability of joint |
| M357 | Hypermobility syndrome |
| M61 | Calcification and ossification of muscle |
| M652 | Calcific tendinitis |
| M653 | Trigger finger |
| M654 | Radial styloid tenosynovitis [de Quervain] |
| M700 | Chronic crepitant synovitis of hand and wrist |
| M720 | Palmar fascial fibromatosis [Dupuytren] |
| M722 | Plantar fascial fibromatosis |
| M724 | Pseudosarcomatous fibromatosis |
| M750 | Adhesive capsulitis of shoulder |
| M751 | Rotator cuff syndrome |
| M753 | Calcific tendinitis of shoulder |
| M754 | Impingement syndrome of shoulder |
| M797 | Fibromyalgia |
| M841 | Nonunion of fracture [pseudarthrosis] |
| M89 | Other disorders of bone |
| M91 | Juvenile osteochondrosis of hip and pelvis |
| M93 | Other osteochondropathies |
| M94 | Other disorders of cartilage |
| M96 | Postprocedural musculoskeletal disorders, not elsewhere classified |
| M99 | Biomechanical lesions, not elsewhere classified |
| Q65 | Congenital deformities of hip |
| Q66 | Congenital deformities of feet |
| Q68 | Other congenital musculoskeletal deformities |
| Q71 | Reduction defects of upper limb |
| Q72 | Reduction defects of lower limb |
| Q73 | Reduction defects of unspecified limb |
| Q74 | Other congenital malformations of limb(s) |
| Q77 | Osteochondrodysplasia with defects of growth of tubular bones and spine |
| Q78 | Other osteochondrodysplasias |
| Q796 | Ehlers-Danlos syndrome |
| Q798 | Other congenital malformations of musculoskeletal system |
| Q87 | Other specified congenital malformation syndromes affecting multiple systems |
| S382 | Traumatic amputation of external genital organs |
| S48 | Traumatic amputation of shoulder and upper arm |
| S58 | Traumatic amputation of forearm |
| S68 | Traumatic amputation of wrist and hand |
| S78 | Traumatic amputation of hip and thigh |
| S88 | Traumatic amputation of lower leg |
| S98 | Traumatic amputation of ankle and foot |
| T05 | Traumatic amputations involving multiple body regions |
| T096 | Traumatic amputation of trunk, level unspecified |
| T116 | Traumatic amputation of upper limb, level unspecified |
| T136 | Traumatic amputation of lower limb, level unspecified |
| T147 | Crushing injury and traumatic amputation of unspecified body region |
| T90 | Sequelae of injuries of head |
| T91 | Sequelae of injuries of neck and trunk |
| T92 | Sequelae of injuries of upper limb |
| T93 | Sequelae of injuries of lower limb |
| T94 | Sequelae of injuries involving multiple and unspecified body regions |
| T95 | Sequelae of burns, corrosions and frostbite |
| T96 | Sequelae of poisoning by drugs, medicaments and biological substances |
| T97 | Sequelae of toxic effects of substances chiefly nonmedicinal as to source |
| T98 | Sequelae of other and unspecified effects of external causes |
| Z440 | Fitting and adjustment of artificial arm (complete)(partial) |
| Z441 | Fitting and adjustment of artificial leg (complete)(partial) |
| Z891 | Acquired absence of hand and wrist |
| Z892 | Acquired absence of upper limb above wrist |
| Z893 | Acquired absence of both upper limbs [any level] |
| Z894 | Acquired absence of foot and ankle |
| Z895 | Acquired absence of leg at or below knee |
| Z896 | Acquired absence of leg above knee |
| Z897 | Acquired absence of both lower limbs [any level, except toes alone] |
| Z898 | Acquired absence of upper and lower limbs [any level] |
| Z899 | Acquired absence of limb, unspecified |
| Z946 | Bone transplant status |
| Z966 | Presence of orthopaedic joint implants |
| Z971 | Presence of artificial limb (complete)(partial) |
| **OTHER NEUROLOGICAL DISEASES** | |
| **Included ICD-10 codes and labels** | |
| B900 | Sequelae of central nervous system tuberculosis |
| D482 | Neoplasm of uncertain or unknown behaviour: Peripheral nerves and autonomic nervous system |
| G041 | Tropical spastic paraplegia |
| G09 | Sequelae of inflammatory diseases of central nervous system |
| G10 | Huntington disease |
| G11 | Hereditary ataxia |
| G12 | Spinal muscular atrophy and related syndromes |
| G13 | Systemic atrophies primarily affecting central nervous system in diseases classified elsewhere |
| G24 | Dystonia |
| G25 | Other extrapyramidal and movement disorders |
| G26 | Extrapyramidal and movement disorders in diseases classified elsewhere |
| G32 | Other degenerative disorders of nervous system in diseases classified elsewhere |
| G37 | Other demyelinating diseases of central nervous system |
| G51 | Facial nerve disorders |
| G52 | Disorders of other cranial nerves |
| G53 | Cranial nerve disorders in diseases classified elsewhere |
| G70 | Myasthenia gravis and other myoneural disorders |
| G71 | Primary disorders of muscles |
| G723 | Periodic paralysis |
| G724 | Inflammatory myopathy, not elsewhere classified |
| G728 | Other specified myopathies |
| G729 | Myopathy, unspecified |
| G73 | Disorders of myoneural junction and muscle in diseases classified elsewhere |
| G80 | Cerebral palsy |
| G81 | Hemiplegia |
| G82 | Paraplegia and tetraplegia |
| G83 | Other paralytic syndromes |
| G90 | Disorders of autonomic nervous system |
| G91 | Hydrocephalus |
| G938 | Other specified disorders of brain |
| G939 | Disorder of brain, unspecified |
| G95 | Other diseases of spinal cord |
| G99 | Other disorders of nervous system in diseases classified elsewhere |
| M471 | Other spondylosis with myelopathy |
| Q00 | Anencephaly and similar malformations |
| Q01 | Encephalocele |
| Q02 | Microcephaly |
| Q03 | Congenital hydrocephalus |
| Q04 | Other congenital malformations of brain |
| Q05 | Spina bifida |
| Q06 | Other congenital malformations of spinal cord |
| Q07 | Other congenital malformations of nervous system |
| Q760 | Spina bifida occulta |
| **Excluded ICD-10 codes and labels** | |
| G130 | Paraneoplastic neuromyopathy and neuropathy |
| G131 | Other systemic atrophy primarily affecting central nervous system in neoplastic disease |
| G251 | Drug-induced tremor |
| G254 | Drug-induced chorea |
| G256 | Drug-induced tics and other tics of organic origin |
| G510 | Bell palsy |
| G732 | Other myasthenic syndromes in neoplastic disease |
| G733 | Myasthenic syndromes in other diseases classified elsewhere |
| G734 | Myopathy in infectious and parasitic diseases classified elsewhere |
| G838 | Other specified paralytic syndromes |
| **OTHER PSYCHIATRIC AND BEHAVIORAL DISEASES** | |
| **Included ICD-10 codes and labels** | |
| F04 | Organic amnesic syndrome, not induced by alcohol and other psychoactive substances |
| F06 | Other mental disorders due to brain damage and dysfunction and to physical disease |
| F07 | Personality and behavioural disorders due to brain disease, damage and dysfunction |
| F09 | Unspecified organic or symptomatic mental disorder |
| F102 | Mental and behavioural disorders due to use of alcohol: Dependence syndrome |
| F106 | Mental and behavioural disorders due to use of alcohol: Amnesic syndrome |
| F107 | Mental and behavioural disorders due to use of alcohol: Residual and late-onset psychotic disorder |
| F112 | Mental and behavioural disorders due to use of opioids: Dependence syndrome |
| F116 | Mental and behavioural disorders due to use of opioids: Amnesic syndrome |
| F117 | Mental and behavioural disorders due to use of opioids: Residual and late-onset psychotic disorder |
| F122 | Mental and behavioural disorders due to use of cannabinoids: Dependence syndrome |
| F126 | Mental and behavioural disorders due to use of cannabinoids: Amnesic syndrome |
| F127 | Mental and behavioural disorders due to use of cannabinoids: Residual and late-onset psychotic disorder |
| F132 | Mental and behavioural disorders due to use of sedatives or hypnotics: Dependence syndrome |
| F136 | Mental and behavioural disorders due to use of sedatives or hypnotics: Amnesic syndrome |
| F137 | Mental and behavioural disorders due to use of sedatives or hypnotics: Residual and late-onset psychotic disorder |
| F142 | Mental and behavioural disorders due to use of cocaine: Dependence syndrome |
| F146 | Mental and behavioural disorders due to use of cocaine: Amnesic syndrome |
| F147 | Mental and behavioural disorders due to use of cocaine: Residual and late-onset psychotic disorder |
| F152 | Mental and behavioural disorders due to use of other stimulants, including caffeine: Dependence syndrome |
| F156 | Mental and behavioural disorders due to use of other stimulants, including caffeine: Amnesic syndrome |
| F157 | Mental and behavioural disorders due to use of other stimulants, including caffeine: Residual and late-onset psychotic disorder |
| F162 | Mental and behavioural disorders due to use of hallucinogens: Dependence syndrome |
| F166 | Mental and behavioural disorders due to use of hallucinogens: Amnesic syndrome |
| F167 | Mental and behavioural disorders due to use of hallucinogens: Residual and late-onset psychotic disorder |
| F172 | Mental and behavioural disorders due to use of tobacco: Dependence syndrome |
| F176 | Mental and behavioural disorders due to use of tobacco: Amnesic syndrome |
| F177 | Mental and behavioural disorders due to use of tobacco: Residual and late-onset psychotic disorder |
| F182 | Mental and behavioural disorders due to use of volatile solvents: Dependence syndrome |
| F186 | Mental and behavioural disorders due to use of volatile solvents: Amnesic syndrome |
| F187 | Mental and behavioural disorders due to use of volatile solvents: Residual and late-onset psychotic disorder |
| F192 | Mental and behavioural disorders due to multiple drug use and use of other psychoactive substances: Dependence syndrome |
| F196 | Mental and behavioural disorders due to multiple drug use and use of other psychoactive substances: Amnesic syndrome |
| F197 | Mental and behavioural disorders due to multiple drug use and use of other psychoactive substances: Residual and late-onset psychotic disorder |
| F50 | Eating disorders |
| F52 | Sexual dysfunction, not caused by organic disorder or disease |
| F60 | Specific personality disorders |
| F61 | Mixed and other personality disorders |
| F62 | Enduring personality changes, not attributable to brain damage and disease |
| F63 | Habit and impulse disorders |
| F68 | Other disorders of adult personality and behaviour |
| F70 | Mild mental retardation |
| F71 | Moderate mental retardation |
| F72 | Severe mental retardation |
| F73 | Profound mental retardation |
| F78 | Other mental retardation |
| F79 | Unspecified mental retardation |
| F80 | Specific developmental disorders of speech and language |
| F81 | Specific developmental disorders of scholastic skills |
| F82 | Specific developmental disorder of motor function |
| F83 | Mixed specific developmental disorders |
| F84 | Pervasive developmental disorders |
| F88 | Other disorders of psychological development |
| F89 | Unspecified disorder of psychological development |
| F95 | Tic disorders |
| F99 | Mental disorder, not otherwise specified |
| **OTHER RESPIRATORY DISEASES** | |
| **Included ICD-10 codes and labels** | |
| B909 | Sequelae of respiratory and unspecified tuberculosis |
| E662 | Extreme obesity with alveolar hypoventilation |
| J60 | Coalworker pneumoconiosis |
| J61 | Pneumoconiosis due to asbestos and other mineral fibres |
| J62 | Pneumoconiosis due to dust containing silica |
| J63 | Pneumoconiosis due to other inorganic dusts |
| J64 | Unspecified pneumoconiosis |
| J65 | Pneumoconiosis associated with tuberculosis |
| J66 | Airway disease due to specific organic dust |
| J67 | Hypersensitivity pneumonitis due to organic dust |
| J684 | Chronic respiratory conditions due to chemicals, gases, fumes and vapours |
| J701 | Chronic and other pulmonary manifestations due to radiation |
| J703 | Chronic drug-induced interstitial lung disorders |
| J704 | Drug-induced interstitial lung disorders, unspecified |
| J84 | Other interstitial pulmonary diseases |
| J92 | Pleural plaque |
| J941 | Fibrothorax |
| J953 | Chronic pulmonary insufficiency following surgery |
| J955 | Postprocedural subglottic stenosis |
| J961 | Chronic respiratory failure |
| J98 | Other respiratory disorders |
| Q33 | Congenital malformations of lung |
| Q34 | Other congenital malformations of respiratory system |
| Z902 | Acquired absence of lung [part of] |
| Z942 | Lung transplant status |
| Z943 | Heart and lungs transplant status |
| Z963 | Presence of artificial larynx |
| **Excluded ICD-10 codes and labels** | |
| J981 | Pulmonary collapse |
| **OTHER SKIN DISEASES** | |
| **Included ICD-10 codes and labels** | |
| L13 | Other bullous disorders |
| L28 | Lichen simplex chronicus and prurigo |
| L301 | Dyshidrosis [pompholyx] |
| L43 | Lichen planus |
| L508 | Other urticaria |
| L581 | Chronic radiodermatitis |
| L85 | Other epidermal thickening |
| Q80 | Congenital ichthyosis |
| Q81 | Epidermolysis bullosa |
| Q821 | Xeroderma pigmentosum |
| Q822 | Mastocytosis |
| Q829 | Congenital malformation of skin, unspecified |
| **Excluded ICD-10 codes and labels** | |
| L432 | Lichenoid drug reaction |
| **PARKINSON AND PARKINSONISM** | |
| **Included ICD-10 codes and labels** | |
| G20 | Parkinson disease |
| G21 | Secondary parkinsonism |
| G22 | Parkinsonism in diseases classified elsewhere |
| G23 | Other degenerative diseases of basal ganglia |
| **Excluded ICD-10 codes and labels** | |
| G210 | Malignant neuroleptic syndrome |
| **PERIPHERAL NEUROPATHY** | |
| **Included ICD-10 codes and labels** | |
| B91 | Sequelae of poliomyelitis |
| G14 | Postpolio syndrome |
| G54 | Nerve root and plexus disorders |
| G55 | Nerve root and plexus compressions in diseases classified elsewhere |
| G56 | Mononeuropathies of upper limb |
| G57 | Mononeuropathies of lower limb |
| G58 | Other mononeuropathies |
| G59 | Mononeuropathy in diseases classified elsewhere |
| G60 | Hereditary and idiopathic neuropathy |
| G628 | Other specified polyneuropathies |
| G629 | Polyneuropathy, unspecified |
| G63 | Polyneuropathy in diseases classified elsewhere |
| M472 | Other spondylosis with radiculopathy |
| M531 | Cervicobrachial syndrome |
| M541 | Radiculopathy |
| **Excluded ICD-10 codes and labels** | |
| G631 | Polyneuropathy in neoplastic disease |
| **PERIPHERAL VASCULAR DISEASE** | |
| **Included ICD-10 codes and labels** | |
| I702 | Atherosclerosis of arteries of extremities |
| I73 | Other peripheral vascular diseases |
| I792 | Peripheral angiopathy in diseases classified elsewhere |
| I798 | Other disorders of arteries, arterioles and capillaries in diseases classified elsewhere |
| **Excluded ICD-10 codes and labels** | |
| I731 | Thromboangiitis obliterans [Buerger] |
| I738 | Other specified peripheral vascular diseases |
| **PROSTATE DISEASES** | |
| **Included ICD-10 codes and labels** | |
| N40 | Hyperplasia of prostate |
| N411 | Chronic prostatitis |
| N418 | Other inflammatory diseases of prostate |
| **SCHIZOPHRENIA AND DELUSIONAL DISEASES** | |
| **Included ICD-10 codes and labels** | |
| F20 | Schizophrenia |
| F22 | Persistent delusional disorders |
| F24 | Induced delusional disorder |
| F25 | Schizoaffective disorders |
| F28 | Other nonorganic psychotic disorders |
| **SLEEP DISORDERS** | |
| **Included ICD-10 codes and labels** | |
| F510 | Nonorganic insomnia |
| F511 | Nonorganic hypersomnia |
| F512 | Nonorganic disorder of the sleep-wake schedule |
| F513 | Sleepwalking [somnambulism] |
| G47 | Sleep disorders |
| **SOLID NEOPLASMS** | |
| **Included ICD-10 codes and labels** | |
| C | Malignant neoplasms |
| D00 | Carcinoma in situ of oral cavity, oesophagus and stomach |
| D01 | Carcinoma in situ of other and unspecified digestive organs |
| D02 | Carcinoma in situ of middle ear and respiratory system |
| D03 | Melanoma in situ |
| D04 | Carcinoma in situ of skin |
| D05 | Carcinoma in situ of breast |
| D06 | Carcinoma in situ of cervix uteri |
| D07 | Carcinoma in situ of other and unspecified genital organs |
| D09 | Carcinoma in situ of other and unspecified sites |
| D320 | Benign neoplasm: Cerebral meninges |
| D321 | Benign neoplasm: Spinal meninges |
| D329 | Benign neoplasm: Meninges, unspecified |
| D330 | Benign neoplasm: Brain, supratentorial |
| D331 | Benign neoplasm: Brain, infratentorial |
| D332 | Benign neoplasm: Brain, unspecified |
| D333 | Benign neoplasm: Cranial nerves |
| D334 | Benign neoplasm: Spinal cord |
| Q85 | Phakomatoses, not elsewhere classified |
| **Excluded ICD-10 codes and labels** | |
| C81 | Hodgkin lymphoma |
| C82 | Follicular lymphoma |
| C83 | Non-follicular lymphoma |
| C84 | Mature T/NK-cell lymphomas |
| C85 | Other and unspecified types of non-Hodgkin lymphoma |
| C86 | Other specified types of T/NK-cell lymphoma |
| C88 | Malignant immunoproliferative diseases |
| C90 | Multiple myeloma and malignant plasma cell neoplasms |
| C91 | Lymphoid leukaemia |
| C92 | Myeloid leukaemia |
| C93 | Monocytic leukaemia |
| C94 | Other leukaemias of specified cell type |
| C95 | Leukaemia of unspecified cell type |
| C96 | Other and unspecified malignant neoplasms of lymphoid, haematopoietic and related tissue |
| **THYROID DISEASES** | |
| **Included ICD-10 codes and labels** | |
| E00 | Congenital iodine-deficiency syndrome |
| E01 | Iodine-deficiency-related thyroid disorders and allied conditions |
| E02 | Subclinical iodine-deficiency hypothyroidism |
| E03 | Other hypothyroidism |
| E05 | Thyrotoxicosis [hyperthyroidism] |
| E062 | Chronic thyroiditis with transient thyrotoxicosis |
| E063 | Autoimmune thyroiditis |
| E065 | Other chronic thyroiditis |
| E07 | Other disorders of thyroid |
| E350 | Disorders of thyroid gland in diseases classified elsewhere |
| E890 | Postprocedural hypothyroidism |
| **Excluded ICD-10 codes and labels** | |
| E035 | Myxoedema coma |
| **VENOUS AND LYMPHATIC DISEASES** | |
| **Included ICD-10 codes and labels** | |
| I780 | Hereditary haemorrhagic telangiectasia |
| I83 | Varicose veins of lower extremities |
| I87 | Other disorders of veins |
| I89 | Other noninfective disorders of lymphatic vessels and lymph nodes |
| I972 | Postmastectomy lymphoedema syndrome |
| Q820 | Hereditary lymphoedema |

NOTE: When all sub-codes within a given ICD-10 code were classified as chronic, the highest possible level of aggregation of the hierarchy was included in the list (e.g. three-digit code for asthma (J45), one-digit code for malignant neoplasms (C), etc.).

**Supplementary Table 2. Description of all variables in the model**

| **Name (Spanish)** | **Name (English)** | **Explanation** |
| --- | --- | --- |
| *edad* | age | Patient age |
| *n urg* | number of emergencies | Number of emergency admissions |
| *n hosp* | number of hospitalizations | Number of hospitalizations |
| *n cx* | number of surgeries | Number of surgeries |
| *n ce med general* | number of outpatient consultations per general practitioner | Number of medical consultations in general medicine |
| *n ce med especializada* | number of outpatient consultations per specialist doctor | Number of medical consultations in specialized medicine |
| *estancia sala* | general hospitalization room stay | Length of stay in the hospital ward |
| *estancia uce* | stay in special care | Length of stay in the emergency intensive care unit |
| *estancia uci* | ICU stay | Length of stay in the intensive care unit |
| *pancreatobiliar* | pancreatobiliary | Diagnosis of pancreatic or biliary disease |
| *glaucoma* | glaucoma | Glaucoma diagnosis |
| *hipertensión* | hypertension | Diagnosis of hypertension |
| *cerebro vascular* | vascular brain | Diagnosis of cerebrovascular disease |
| *vertigo y alteraciones auditivas* | vertigo and hearing disturbances | Diagnosis of vertigo or hearing disorders |
| *bradicardias y enfermedades de la conduccion* | bradycardias and conduction diseases | Diagnosis of bradycardia or conduction disease |
| *otras genitourinarias* | other genitourinary | Diagnosis of other genitourinary diseases |
| *depresion y alteraciones del animo* | depression and mood disorders | Diagnosis of depression or mood disorders |
| *EPOC* | COPD | Diagnosis of chronic obstructive pulmonary disease |
| *enfermedad renal cronica* | chronic kidney disease | Diagnosis of chronic kidney disease |
| *enfermedades de la tiroides* | thyroid diseases | Diagnosis of thyroid diseases. |
| *otras enfermedades digestivas* | other digestive diseases | Diagnosis of other digestive diseases |
| *hematologicas* | hematological | Diagnosis of blood-related diseases. |
| *alteraciones otorrinolaringologicas* | otorhinolaryngological disorders | Diagnosis of diseases related to the ears, nose and throat |
| *obesidad* | obesity | Diagnosis of obesity |
| *enfermedades de la prostata* | prostate diseases | Diagnosis of prostate diseases. |
| *esquizofrenia* | schizophrenia | Diagnosis of schizophrenia |
| *vascular periferica* | peripheral vascular | Diagnosis of peripheral vascular disease |
| *alteraciones de la agudeza visual* | alterations in visual acuity | Diagnosis of visual acuity alterations |
| *cromosómicas* | chromosomal | Diagnosis of chromosomal abnormalities |
| *osteoporosis* | osteoporosis | Diagnosis of osteoporosis |
| *infecciones cronicas* | chronic infections | Diagnosis of chronic infections |
| *anemia* | anemia | Diagnosis of anemia |
| *somatomorfos* | somatomorphs | Diagnosis of somatoform disorders |
| *otras enfermedades oculares* | other eye diseases | Diagnosis of other eye diseases. |
| *colitis y gastrointestinales inferiores* | colitis and lower gastrointestinal | Diagnosis of colitis or other lower gastrointestinal diseases |
| *artrosis* | osteoarthritis | Diagnosis of osteoarthritis |
| *enfermedad isquemica cardiaca* | ischemic heart disease | Diagnosis of ischemic heart disease |
| *otras respiratorias* | other respiratory | Diagnosis of other respiratory diseases |
| *cataratas* | waterfalls | Cataract diagnosis |
| *hepatopatia cronica* | chronic liver disease | Diagnosis of chronic liver disease |
| *otras neurologicas* | other neurological | Diagnosis of other neurological diseases |
| *otras psiquiatricas* | other psychiatric | Diagnosis of other psychiatric illnesses |
| *valvulares* | valves | Diagnosis of valvular heart disease |
| *otras dermatologicas* | other dermatological | Other dermatological conditions |
| *autoinmunes* | autoimmune | Autoimmune conditions |
| *trastornos del sueño* | sleep disorders | Sleep disorders |
| *enfermedad venosa y linfatica* | venous and lymphatic disease | Venous and lymphatic disease |
| *migraña y síndromes faciales dolorosos* | migraine and facial pain syndromes | Migraine and facial pain syndromes |
| *dislipidemia* | dyslipidemia | Dyslipidemia (abnormal levels of lipids in the blood) |
| *esclerosis multiple* | multiple sclerosis | Multiple sclerosis |
| *demencias* | dementias | Dementia |
| *alergia* | allergy | Allergy |
| *asma* | asthma | Asthma |
| *úlceras* | ulcers | Ulcers |
| *enfermedad inflamatoria intestinal* | inflammatory bowel disease | inflammatory bowel disease |
| *neuropatias perifericas* | peripheral neuropathies | Peripheral neuropathy |
| *epilepsia* | epilepsy | Epilepsy |
| *artropatías inflamatorias* | inflammatory arthropathies | Inflammatory joint diseases |
| *lumbalgia cronica* | chronic low back pain | Chronic low back pain |
| *otras enfermedades cardiovasculares* | other cardiovascular diseases | Other cardiovascular diseases |
| *parkinson* | parkinson | Parkinson's disease |
| *cáncer* | cancer | Cancer |
| *fibrilacion auricular* | atrial fibrillation | atrial fibrillation |
| *diabetes* | diabetes | Diabetes |
| *falla cardiaca* | heart failure | Heart failure |
| *gastrointestinales superiores* | upper gastrointestinal | Upper gastrointestinal disorders |
| *hechos otros* | other facts | Other factors |
| *fact estancias* | fact stays | Hospital stays |
| *fact ayudas dx* | fact aid dx | Diagnostic aids |
| *fact interconsultas* | fact interconsultations | Interconsultations |
| *fact medicamentos insumos* | fact medicines supplies | Medications and supplies |
| *fact otros conceptos* | fact other concepts | Other concepts |
| *fact procedimientos paquetes qx* | fact procedures qx packages | Procedures and surgery packages |
| *hecho rondas* | done rounds | medical rounds |
| *fact banco sangre* | fact blood bank | blood bank |
| *fact planta oxigeno* | fact plant oxygen | Oxygen supply |
| *fact consulta externa* | fact external consultation | External consultations |
| *fact salud oral* | fact oral health | Oral health |
| *hecho total* | complete fact | Total cost of health care |
| *peso* | weight | Weight |
| *talla* | size | Height |
| *saturacion oxigeno* | oxygen saturation | Oxygen saturation |
| *perímetro muslo* | thigh perimeter | thigh circumference |
| *perímetro cintura* | waist circumference | Waist circumference |
| *pliegue tríceps* | triceps fold | Triceps skin fold thickness |
| *pliegue abdomen* | abdomen fold | Abdominal skinfold thickness |
| *pliegue muslo* | thigh fold | Thigh skinfold thickness |
| *presión arterial sistólica* | systolic blood pressure | Systolic blood pressure |
| *presión arterial diastólica* | diastolic blood pressure | Diastolic blood pressure |
| *frecuencia cardíaca en reposo* | resting heart rate | Resting heart rate |
| *auto-calificacion nivel de ejercicio* | exercise level self-rating | Self-assessed exercise level |
| *constantes* | constants | Vital signs (for example, temperature, heart rate, respiratory rate, blood pressure) |
| *mets -índice metabólico* | mets -metabolic index | Metabolic equivalent of task (a measure of physical activity) |
| *vo2 - máxima cantidad de oxígeno* | vo2 - maximum amount of oxygen | Maximum oxygen consumption during exercise |
| *índice de fragilidad groningen* | groningen fragility index | Groningen Frailty Index (a measure of frailty in older adults) |
| *tiempo segundos monopodal* | time seconds single leg | Single-leg balance time (a measure of balance ability) |
| *tiempo segundos 5metros* | time seconds 5 meters | Time to walk 5 meters |
| *tiempo con el diagnóstico* | time with diagnosis | Time since diagnosis |
| *glicemia* | glycemia | blood glucose level |
| *hemoglobina glicada* | glycated hemoglobin | Glycosylated hemoglobin level (a measure of long-term blood sugar control) |
| *tiempo con el diagnóstico2* | time with diagnosis2 | Time since diagnosis (alternative measure) |
| *cuantos cigarrillos dia* | how many cigarettes a day | Number of cigarettes smoked per day. |
| *años de consumo* | years of consumption | Years of smoking history |
| *lipoproteina hdl* | hdl lipoprotein | HDL cholesterol |
| *colesterol total* | total cholesterol | Total blood cholesterol level |
| *triglicéridos* | triglycerides | Blood triglyceride level |
| *creatinina 1 consulta* | creatinine 1 consultation | Blood creatinine level during the first medical consultation |
| *tasa de filtración glomerular tfg* | glomerular filtration rate tfg | Glomerular filtration rate, a measure of kidney function |
| *microalbuminuria* | microalbuminuria | A condition in which small amounts of the protein albumin are found in the urine, which may be a sign of kidney damage. |
| *tsh* | tsh | Thyroid-stimulating hormone level in the blood |
| *creatinina 2 consulta* | creatinine 2 consultation | Blood creatinine level during the second medical consultation |
| *tasa de filtración glomerular tfg3* | glomerular filtration rate tfg3 | Glomerular filtration rate measured during the third medical visit |
| *cambio de tfg* | change of tfg | Change in glomerular filtration rate between the first and third medical visit |
| *estado vital* | vital status | Vital status, whether the patient is alive or deceased |
| *sexo M* | sex M | Patient's gender, with "M" indicating male |
| *calificacion indicefragilidad Normal* | Normal indicefragility rating | Frailty assessment, with "Normal" indicating no significant frailty |
| *calificación indicefragilidad Frágil* | Fragile indicefragility rating | Frailty assessment, with “Fragile” indicating significant frailty |
| *calificación apoyo monopodal Aceptable* | single-leg support rating Acceptable | Balance rating, with "Acceptable" indicating good balance |
| *calificacion apoyo monopodal Malo...Fragil* | single leg support rating Bad Fragile | Balance assessment, with "Poor... Fragile" indicating poor and fragile balance |
| *calificacion apoyo monopodal Bueno* | single-leg support rating Good | Balance rating, with "Good" indicating excellent balance |
| *calificación velocidad Deficiente* | Poor speed rating | Assessment of walking speed, with "Poor" indicating slow walking speed |
| *calificación velocidad Malo...Frágil* | rating speed Bad Fragile | Assessment of gait speed, with "Poor...Fragile" indicating poor and fragile gait speed |
| *calificación velocidad Aceptable* | Acceptable speed rating | Gait speed assessment, with "Acceptable" indicating good gait speed |
| *calificación velocidad Bueno* | speed rating Good | Gait speed assessment, with "Good" indicating excellent gait speed |
| *indice tobillo brazo X0.91...1.30.Normal* | ankle arm index X0 91 1 30 Normal | Ankle-brachial index, a measure of peripheral arterial disease, where "Normal" indicates no significant disease |
| *indice tobillo brazo X0.41...0.90.Enfermedad.arterial.periférica.leve.a.moderada* | ankle arm index X0 41 0 90 Mild to moderate peripheral arterial disease | Ankle-brachial index, with "Mild to moderate peripheral arterial disease" indicating mild to moderate peripheral arterial disease |
| *indice tobillo brazo X.0.4.EAP.Grave* | index ankle arm X 0 4 PAD Severe | Ankle-brachial index, with "PAD.Severe" indicating severe peripheral arterial disease |
| *diabetes mellitus Si* | diabetes mellitus Yes | If the patient has diabetes mellitus, with "Yes" indicating that the patient has the condition |
| *tipo diabetes mellitus II* | type II diabetes mellitus | Type of diabetes mellitus, where "II" indicates type 2 diabetes |
| *tipo diabetes mellitus I* | type I diabetes mellitus | Type of diabetes mellitus, where "I" indicates type 1 diabetes |
| *es insulinarequisito Si* | is insulin required Yes | If the patient requires insulin for diabetes control, with "Yes" indicating that the patient requires insulin |
| *control diabetes controlada* | controlled diabetes control | If the patient's diabetes is controlled, with "Controlled" indicating that the condition is under control |
| *control diabetes No.controlada* | control diabetes Uncontrolled | If the patient's diabetes is not controlled, with "Not controlled" indicating that the condition is not under control |
| *tiene hta si* | has hta yes | Indicates whether the patient has hypertension or not |
| *control hta Controlada* | Controlled tool control | Indicates whether the patient's hypertension is controlled or not |
| *control hta No.controlada* | tool control Not controlled | Indicates whether the patient's hypertension is uncontrolled or not |
| *epoc bodex Leve* | mild bodex epoc | Indicates whether the patient has chronic obstructive pulmonary disease (COPD) of mild severity |
| *epoc bodex Moderada* | bodex epoc Moderate | Indicates whether the patient has chronic obstructive pulmonary disease (COPD) of moderate severity |
| *epoc bodex Grave* | epoc bodex Grave | Indicates if the patient has chronic obstructive pulmonary disease (COPD) of severe severity |
| *enfermedad coronaria IAM* | coronary heart disease AMI | Indicates whether the patient has had a myocardial infarction (heart attack) |
| *enfermedad coronaria Angina.inestable* | coronary heart disease unstable angina | Tells if the patient has unstable angina (chest pain or discomfort) caused by reduced blood flow to the heart |
| *enfermedad coronaria Angina.estable* | coronary heart disease stable angina | Indicates whether the patient has stable angina (chest pain or discomfort) caused by physical activity or emotional stress |
| *insuficiencia cardiaca Controlada* | Controlled heart failure | Indicates whether the patient's heart failure is controlled or not |
| *insuficiencia cardiaca No.controlada* | Uncontrolled heart failure | Indicates whether the patient's heart failure is uncontrolled or not. |
| *valvulopatía Si* | valve disease Yes | Indicates if the patient has a heart valve disorder |
| *arritmia o paciente con dispositivo Si* | arrhythmia or patient with device Yes | Indicates whether the patient has an arrhythmia (irregular heartbeat) or a device to monitor heart rhythm |
| *sufre de alguna enfermedad cardiovascular Si* | suffer from some cardiovascular disease Yes | Indicates if the patient suffers from any cardiovascular disease |
| *tabaquismo Si* | smoking Yes | Indicates if the patient is currently a smoker |
| *tabaquismo Ex.fumador* | smoking Ex-smoker | Indicates if the patient is an ex-smoker |
| *clasificación de framinghan Bajo.riesgo* | framinghan classification Low risk | Indicates whether the patient is at low risk of developing cardiovascular disease according to the Framingham score |
| *clasificación de framinghan Alto.riesgo* | framinghan classification High risk | Indicates whether the patient is at high risk of developing cardiovascular disease according to the Framingham score |
| *estadio de la enfermedad renal Estadio.2* | stage of kidney disease Stage 2 | Indicates the stage of the patient's kidney disease (stage 2) |
| *estadio de la enfermedad renal Estadio.3A* | stage of kidney disease Stage 3A | Indicates the stage of the patient's kidney disease (stage 3a) |
| *estadio de la enfermedad renal Estadio.1* | stage of kidney disease Stage 1 | Indicates the stage of the patient's kidney disease (stage 1) |
| *estadio de la enfermedad renal Estadio.3B* | stage of kidney disease Stage 3B | Indicates the stage of the patient's kidney disease (stage 3b) |
| *estadio de la enfermedad renal Estadio.4* | stage of kidney disease Stage 4 | Indicates the stage of the patient's kidney disease (stage 4) |
| *estadio de la enfermedad renal Estadio.5* | stage of kidney disease Stage 5 | Indicates the stage of the patient's kidney disease (stage 5) |
| *clase funcional Clase.funcional.2B* | functional class Functional class 2B | Indicates the functional class of the patient with heart failure ( |
| *clase funcional Clase.funcional.2B* | functional class Functional class 2B | Class IIb functional capacity of the heart |
| *clase funcional Clase.funcional.4* | functional class Functional class 4 | Class IV functional capacity of the heart |
| *clase funcional Clase.funcional.2A* | functional class Functional class 2A | Class IIa functional capacity of the heart |
| *clase funcional Clase.funcional.3* | functional class Functional class 3 | Class III functional capacity of the heart |
| *clase funcional Clase.funcional.1* | functional class Functional class 1 | Class I functional capacity of the heart |
| *úlcera de pie diabético Si* | diabetic foot ulcer Yes | If the patient has a foot ulcer related to diabetes mellitus. |

**Supplementary table 3. Prevalence of Chronic Disease Categories Distribution**

| Pancreato-biliary disease, n (%) | 5 (0.1) |
| --- | --- |
| Glaucoma, N (%) | 59 (1.2) |
| Hypertension, n (%) | 3267 (67.4) |
| cerebrovascular, n (%) | 472 (9.7) |
| Vertigo and hearing disturbances, n (%) | 286 (5.9) |
| Bradycardias and conduction diseases, n (%) | 36 (0.7) |
| Other genitourinary, n (%) | 282 (5.8) |
| Depression and mood disturbances, n (%) | 990 (20.4) |
| COPD, N (%) | 940 (19.4) |
| Chronic kidney disease, n (%) | 660 (13.6) |
| Thyroid diseases, n (%) | 808 (16.7) |
| Other digestive diseases, n (%) | 23 (0.5) |
| Hematological, n (%) | 24 (0.5) |
| Otorhinolaryngological disorders, n (%) | 97 (2.0) |
| Obesity, n (%) | 566 (11.7) |
| Prostate diseases, n (%) | 222 (4.6) |
| Schizophrenia, n (%) | 95 (2.0) |
| Peripheral vascular, n (%) | 38 (0.8) |
| Alterations in visual acuity, n (%) | 17 (0.4) |
| Chromosomal, n (%) | 5 (0.1) |
| Osteoporosis, n (%) | 424 (8.8) |
| Chronic infections, n (%) | 45 (0.9) |
| Anemia, n (%) | 136 (2.8) |
| Somatoform, n (%) | 808 (16.7) |
| Other ophthalmological diseases, n (%) | 31 (0.6) |
| Colitis and lower gastrointestinal, n (%) | 248 (5.1) |
| osteoarthritis, n (%) | 798 (16.5) |
| Ischemic heart disease, n (%) | 789 (16.3) |
| Other respiratory, n (%) | 26 (0.5) |
| Cataracts, n (%) | 16 (0.3) |
| Other neurological, n (%) | 220 (4.5) |
| other psychiatric, n (%) | 317 (6.5) |
| Other dermatological, n (%) | 76 (1.6) |
| Autoimmune, n (%) | 186 (3.8) |
| Sleep disorders, n (%) | 345 (7.1) |
| Venous and lymphatic disease, n (%) | 282 (5.8) |
| Migraine and painful facial syndromes, n (%) | 251 (5.2) |
| Dyslipidemia, n (%) | 2057 (42.5) |
| Multiple Sclerosis, n (%) | 5 (0.1) |
| Dementias, n (%) | 443 (9.1) |
| Allergy, n (%) | 141 (2.9) |
| Asthma, n (%) | 87 (1.8) |
| Chronic Ulcers, n (%) | 59 (1.2) |
| Inflammatory Bowel Disease, n (%) | 16 (0.3) |
| Peripheral Neuropathies, n (%) | 271 (5.6) |
| Epilepsy, n (%) | 213 (4.4) |
| Inflammatory Arthropathies, n (%) | 157 (3.2) |
| Chronic Lumbago, n (%) | 152 (3.1) |
| Other Cardiovascular Diseases, n (%) | 120 (2.5) |
| Parkinson, N (%) | 104 (2.1) |
| Cancer, n (%) | 185 (3.8) |
| Atrial fibrillation, n (%) | 350 (7.2) |
| Diabetes, n (%) | 2123 (43.8) |
| Other Cardiac Diseases, n (%) | 545 (11.2) |
| Upper gastrointestinal, n (%) | 268 (5.5) |

**Supplementary Table 4. DeLong test for all models**

| Outcome | Model 1 | Model 2 | AUC Model 1 | AUC Model 2 | Z statistic | p-value |
| --- | --- | --- | --- | --- | --- | --- |
| Hospitalization | Elastic Net | XGBoost | 0,95156 | 0,962524 | -4,81298 | **0,000001** |
| Hospitalization | Elastic Net | Neural Network | 0,95156 | 0,93185 | 2,632704 | **0,008471** |
| Hospitalization | XGBoost | Neural Network | 0,962524 | 0,93185 | 4,246518 | **0,000022** |
| Emergency Room Consultation | Elastic Net | XGBoost | 0,979743 | 0,977232 | 0 | 1 |
| Emergency Room Consultation | Elastic Net | Neural Network | 0,979743 | 0,975742 | 0 | 1 |
| Emergency Room Consultation | XGBoost | Neural Network | 0,977232 | 0,975742 | 0 | 1 |
| Mortality | Elastic Net | XGBoost | 0,883282 | 0,896328 | -0,50985 | 0,610154 |
| Mortality | Elastic Net | Neural Network | 0,883282 | 0,886035 | -0,10536 | 0,916092 |
| Mortality | XGBoost | Neural Network | 0,896328 | 0,886035 | 0,404723 | 0,685681 |

**Supplementary Figure 1. Importance of Variables in the Mortality Prediction Model with Elastic-Net Regression**

**
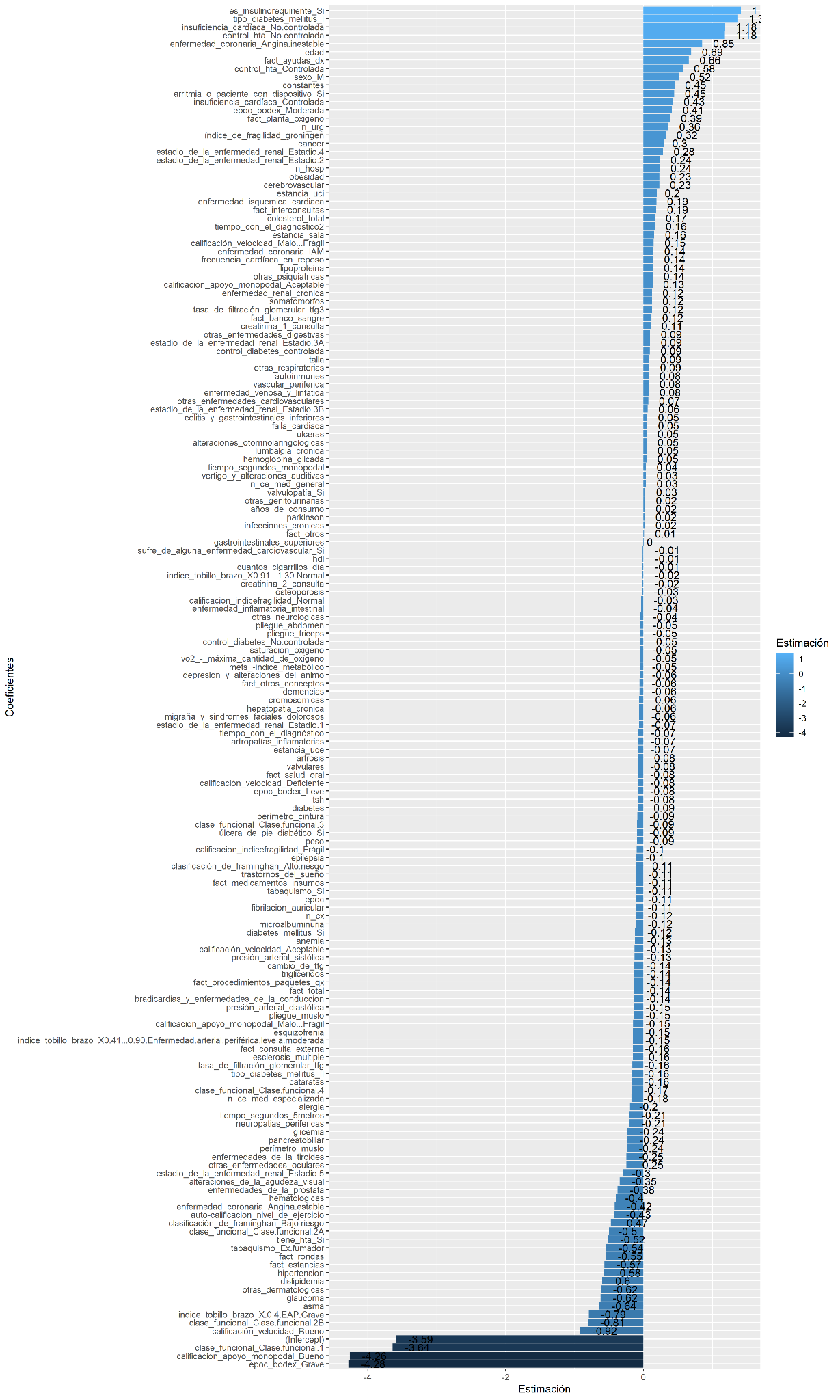
**

**Supplementary Figure 2. Importance of Mortality Prediction Model Variables with XGBoost**

**
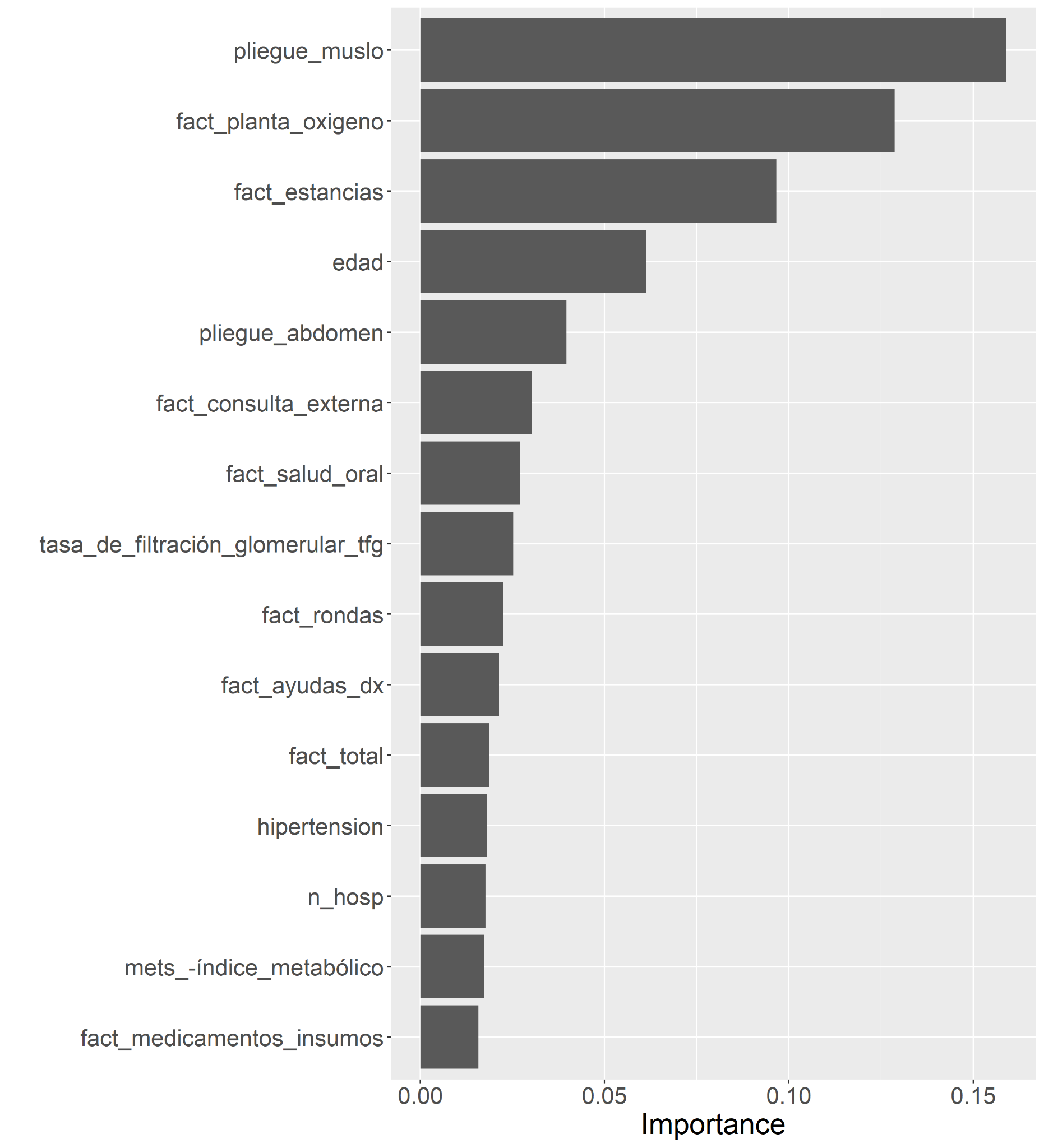
**

**Figure 9. Importance of Variables in the Hospitalization Prediction Model with Elastic-net**

**
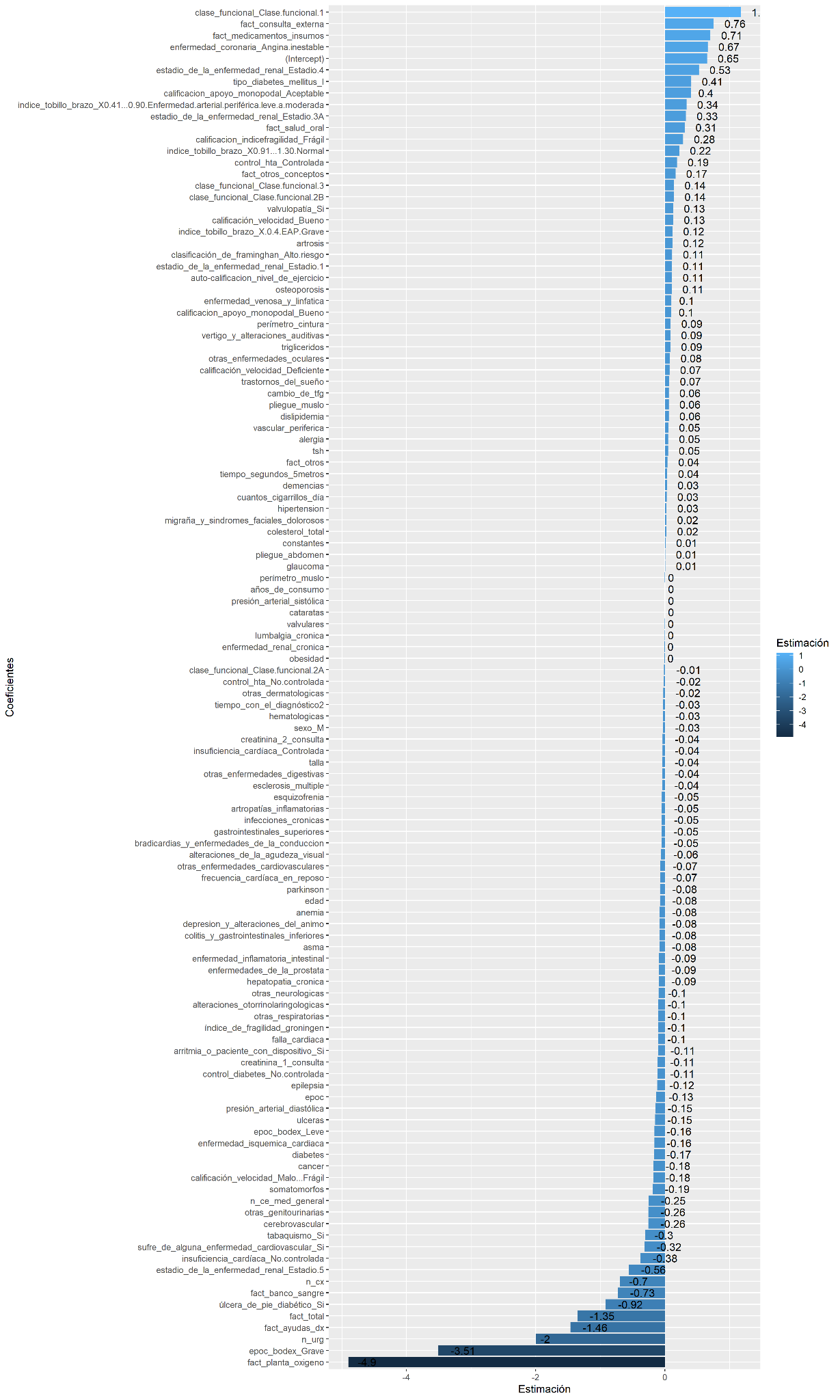
**

**Supplementary Figure 3. Importance of the variables of the hospitalization prediction model with XGBoost**

**
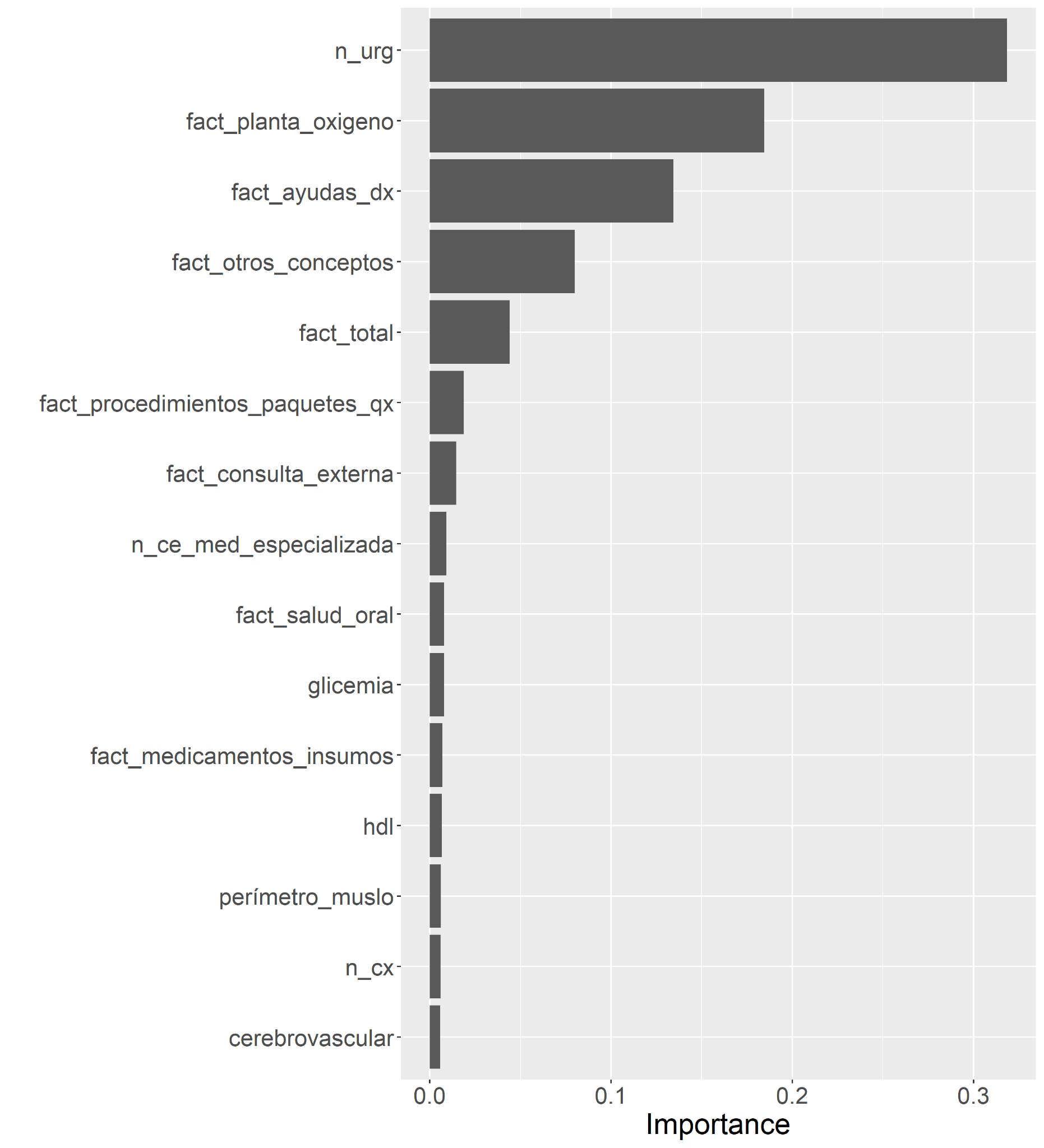
**

**Supplementary Figure 4. Importance of the variables of the prediction model for emergency consultations with Elastic-net.**

**
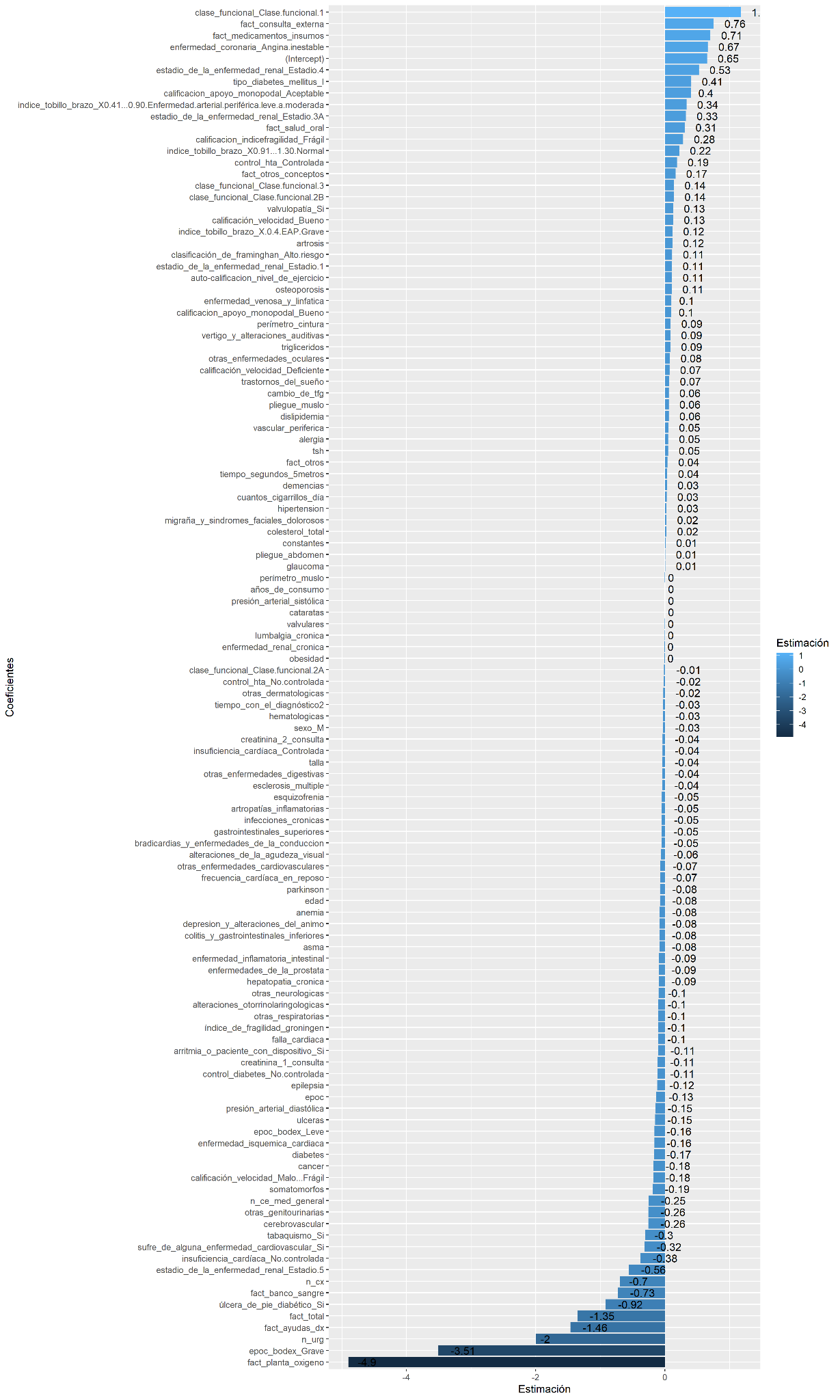
**

**Supplementary Figure 5. Importance of the variables of the prediction model for emergency room visits with XGBoost.**

**
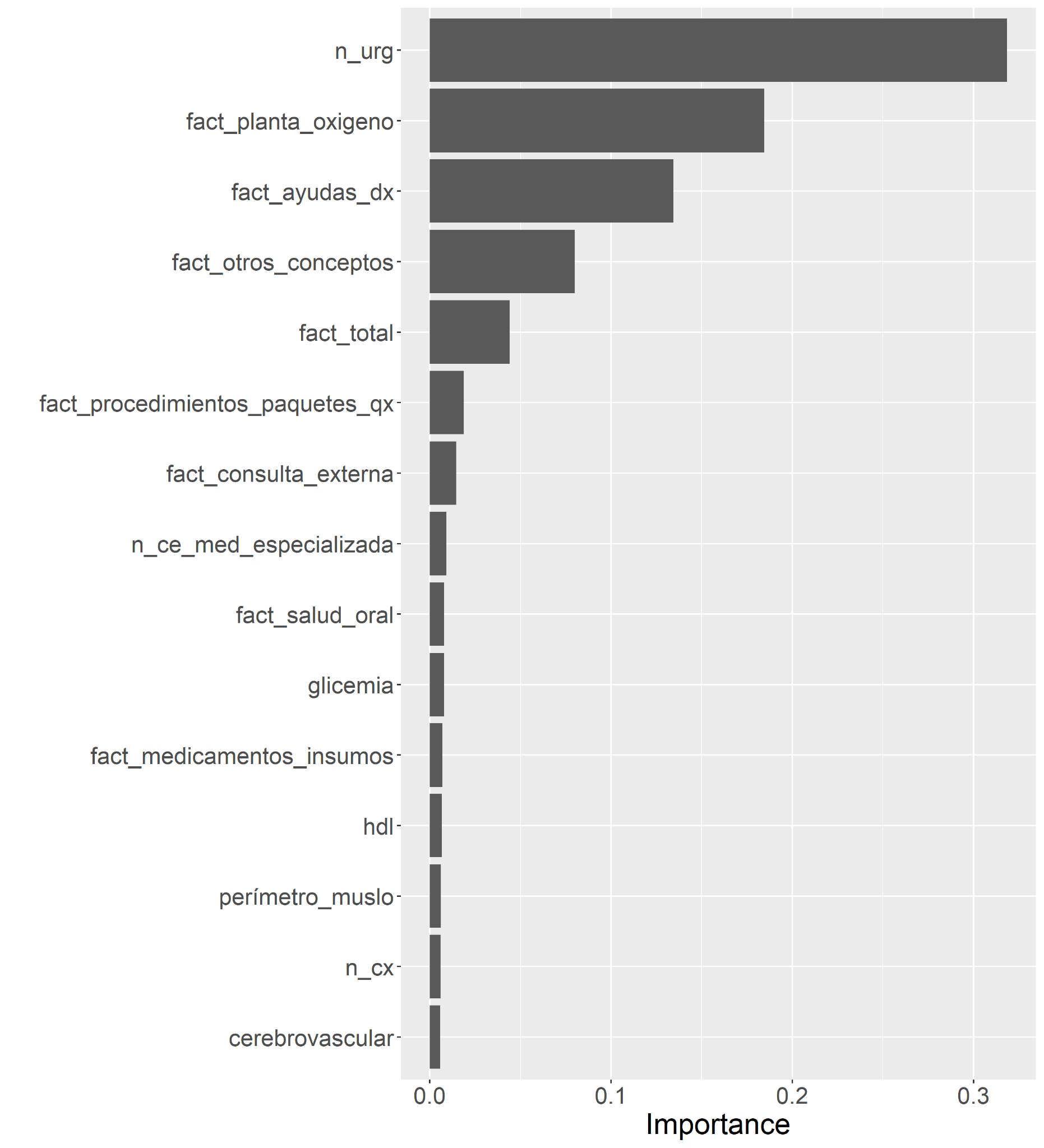
**

**Supplementary Figure 6. Architecture of the Artificial Neural Network (ANN)**


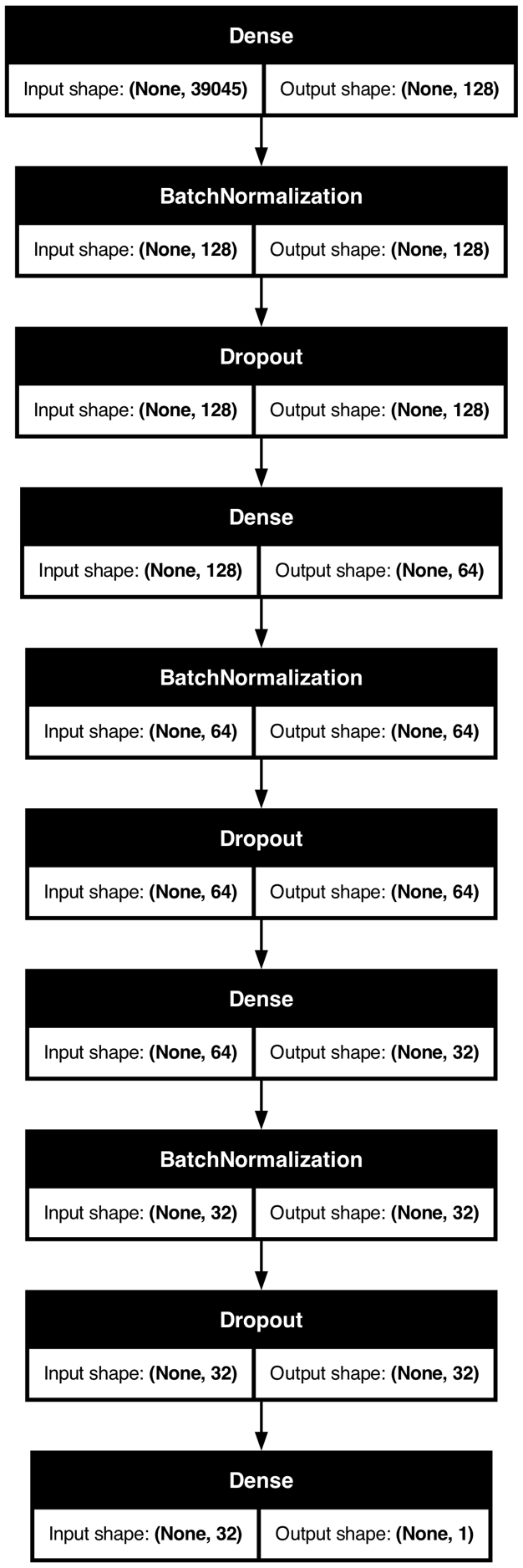

Supplement: Supplementary file 1 — (DOCX 1.90 MB) [file 10916_2025_2140_MOESM1_ESM.docx]
